# Power Analysis for the Wald, LR, Score, and Gradient Tests in a Marginal Maximum Likelihood Framework: Applications in IRT

**DOI:** 10.1007/s11336-022-09883-5

**Published:** 2022-08-27

**Authors:** Felix Zimmer, Clemens Draxler, Rudolf Debelak

**Affiliations:** 1 University of Zurich, Zurich, Switzerland; 2 The Health and Life Sciences University, Hall in Tirol, Austria

**Keywords:** marginal maximum likelihood, item response theory, power analysis, Wald test, score test, likelihood ratio, gradient test

## Abstract

The Wald, likelihood ratio, score, and the recently proposed gradient statistics can be used to assess a broad range of hypotheses in item response theory models, for instance, to check the overall model fit or to detect differential item functioning. We introduce new methods for power analysis and sample size planning that can be applied when marginal maximum likelihood estimation is used. This allows the application to a variety of IRT models, which are commonly used in practice, e.g., in large-scale educational assessments. An analytical method utilizes the asymptotic distributions of the statistics under alternative hypotheses. We also provide a sampling-based approach for applications where the analytical approach is computationally infeasible. This can be the case with 20 or more items, since the computational load increases exponentially with the number of items. We performed extensive simulation studies in three practically relevant settings, i.e., testing a Rasch model against a 2PL model, testing for differential item functioning, and testing a partial credit model against a generalized partial credit model. The observed distributions of the test statistics and the power of the tests agreed well with the predictions by the proposed methods in sufficiently large samples. We provide an openly accessible R package that implements the methods for user-supplied hypotheses.

**Supplementary Information:**

The online version contains supplementary material available at 10.1007/s11336-022-09883-5.

When we draw inferences from models in item response theory (IRT), interpretations are only valid to the degree to which the model is an adequate representation of the observed data (Embretson & Reise 2000). For example in educational assessments, gathering evidence of model fit is generally recommended (American Educational Research Association et al., 2014) as misfits can have varying degrees of practical consequences (Köhler & Hartig, 2017). Model interpretations may decide pass–fail decisions of a test (Sinharay & Haberman, 2014) or even inform educational policy making (van Rijn et al., 2016). Model misfits can be observed at many levels; we can consider more broad misfit, such as applying a wrong type of IRT model (Yen, 1981), or more specific misfits, when interpretations can, for example, carry bias against ethnic groups (Morales et al., 2000). One particularly well-studied local misfit is differential item functioning (DIF), when items are not measuring the same construct for all test takers (Holland & Wainer, 1993). To motivate the use of power analysis in IRT, we first discuss the problem of testing model fit.

*Testing Model Fit*

The tests based on the Wald, likelihood ratio (LR), and score statistics are considered as a standard for large-sample inference (Agresti, 2002; Buse, 1982; Rao, 2005). They are established in IRT for a variety of hypothesis tests, ranging from testing overall model fit to testing hypotheses about specific parameter values (Glas & Verhelst, 1995). The Wald test (Wald, 1943) is typically used for DIF testing (Glas, 2016). The LR test (Silvey, 1959) is known for testing different overall models against each other (Bock & Lieberman, 1970), but can also be used to test for DIF (Irwin et al., 2010; Thissen et al., 1986) . The score test (Rao, 1948) is very flexible since the calculation of the score statistic is less complex than for the Wald and LR statistics (Guastadisegni et al., 2021). There is ample research on utilizing score statistics to test for general model fit (Haberman, 2006), model violations based on item characteristic curves, violations of local independence (Glas, 1999; Glas & Falcón, 2003; Liu & Maydeu-Olivares, 2013), person fit (Glas & Dagohoy, 2007), or DIF (Glas, 1998). In a more general framework, score-based statistics can be used to test for parameter invariance along the values of a covariate (Merkle et al., 2014; Merkle & Zeileis, 2013).

Recently, the gradient statistic was introduced as a complement to the Wald, LR, and score statistics (Lemonte, 2016 ; Terrell, 2002). It is asymptotically equivalent to the other three statistics without uniform superiority of one of them (Lemonte & Ferrari, 2012). It is easier to compute in many instances because it does not require the estimation of an information matrix. Draxler et al. (2020) showcased an application in IRT where the gradient test provided a comparatively higher power than the other statistics. Outside of IRT, the gradient statistic has been recently applied, for example, in multidimensional signal detection (Ciuonzo et al., 2016), segmented regression (Muggeo, 2017), or symmetric linear regression (Medeiros & Ferrari, 2017). One potential weakness of the four mentioned statistics is that significant test results are not always informative regarding the source of the misfit. Estimating generalized residuals is one approach to further determine the causes of misfit (Haberman & Sinharay, 2013).

*The Role of Power Analysis*

Statistical power is defined as the probability of a test to reject the null hypothesis in case that an alternative hypothesis is true (Cohen, 1988). Estimating and reporting the statistical power has been firmly established as a recommended procedure in empirical research (National Academies of Sciences, Engineering, and Medicine, 2019). It depends on many factors, including sample size, effect size, and the study design. Before data collection, we can determine how many participants are required to achieve a certain level of power. For model fitting purposes, power analysis answers the question of how many participants are needed to reject a wrongly assumed model with a predetermined desired probability. When the sample size is fixed, power analysis can also be used to find the size of an effect that can be detected with sufficient power. After data collection, it provides an additional perspective on the observed result, but should be interpreted with caution regarding the overall study design (Cumming, 2014).

Both over- and underpowered study designs should be avoided. Firstly, as the definition of power indicates, studies with too little power are more likely to miss effects that are actually present. Moreover, significant results from studies with low power are less likely to represent actual effects (Button et al., 2013). Low power in general has been a major discussion point in the course of the recent replication crisis (Cumming, 2014; Dwyer et al., 2018). Overpowered studies, on the other hand, can imply a waste of expensive resources such as money, time, or effort (Jak et al., 2021). There are many available approaches for power analysis, for example, for multilevel modeling (Snijders, 2005), structural equation models (Kyriazos, 2018), or ANOVAs (Lakens & Caldwell, 2021).

When applied with IRT models, power analysis can help with the evaluation of study designs for psychological and educational assessments. For example, keeping all other study design parameters equal, power analysis allows an assessment of whether 10 or only 5 items are sufficient to compare the mean ability in two groups (Glas et al., 2009). Or, as another example, one can evaluate whether raising the number of items or the number of participants is the more efficient option to increase the power (Holman et al., 2003). Power analysis is considered critical for designing clinical trials involving patient reported outcomes that are modeled using IRT (Hu et al., 2021). An oversized sample can raise ethical issues, e.g., by delaying the potentially helpful outcome through longer follow-up periods (Blanchin et al., 2015). Also, the possibility of exposing patients to inappropriate medical strategies makes power analysis importantly needed (Hardouin et al., 2012). In educational assessment, sample size planning and power analysis can inform decisions on retaining, modifying, or removing items. Because item development costs time and money, power analysis can help make such decisions at early stages of research. If items need to be changed or removed at later stages, sub-scales might not represent the construct adequately anymore (Köhler & Hartig, 2017). Effect size plays an important role here, since the usually large sample sizes in educational assessments lead to a detection of misfit for many hypotheses (Jodoin & Gierl, 2001). However, power for testing local hypotheses (e.g., a group difference on a single item) is usually lower than for global hypothesis tests (e.g., regarding overall fit of an IRT model) (Jobst et al., 2021; Wang & Rhemtulla, 2021). In the area of psychological diagnostics, another central application of IRT, using misspecified models can lead to misinterpretation of person characteristics which can in turn lead to discrimination against individuals, e.g., in the measurement of psychopathy (Reise & Waller, 2003).

*Existing Methods for Power Analysis*

One approach to power analysis in IRT is referring to rules of thumb, e.g., on the number of persons required for applying a specific IRT model. For example, at least 250 respondents have been suggested for estimating the parameters of the partial credit model (PCM, Masters, 1982; de Ayala, 2009). However, those should not be interpreted as hard rules because they are relatively imprecise (de Ayala, 2009). In spite of the relevance of power analysis for IRT, exact methods of power analysis are rarely presented. As one exception, Maydeu-Olivares and Montaño (2013) have described a method of power analysis for some tests of contingency tables, such as the $M_2$ test. Draxler (2010) and Draxler and Alexandrowicz (2015) provided formulas to calculate the power or sample size of the Wald, LR, and score tests in the context of exponential family models and conditional maximum likelihood (CML) estimation. For the gradient test, Lemonte and Ferrari (2012) provided a comparison of the power for all four statistics again for exponential family models. Two examples of exponential family models in IRT are the Rasch model (Rasch, 1960) and the PCM, in which the item parameters represent the item difficulties. Several estimation methods are available for this family of models, in particular, CML and marginal maximum likelihood (MML) estimation (Baker & Kim, 2004). The advantage of the CML approach lies in the reliance on the participants’ overall test score as a sufficient statistic for their underlying ability. However, some information can be lost using CML compared to MML estimation, especially with smaller numbers of items (Eggen, 2000).

However, models that do not belong to an exponential family cannot be estimated by CML because they do not offer a sufficient statistic. Examples are models that extend the Rasch model by a slope parameter (two-parameter logistic model, 2PL) or a guessing parameter (three-parameter logistic model, 3PL; Birnbaum 1968). These more complex IRT models are commonly applied. In the Trends in International Mathematics and Science Study (TIMSS, Martin et al., 2020), for example, the scaling model was changed from a Rasch to a 3PL model in 1999. More recently, the methodology in the Program for International Student Assessment (PISA) was changed from a Rasch to a 2PL model and from a PCM to a generalized partial credit model (GPCM, Muraki, 1992; OECD 2017; Robitzsch et al. 2020). An approach applicable to MML estimation would allow either hypotheses to refer to more complex models and enable, e.g., a power analysis for a test of a Rasch model against a 2PL model.

*The Present Contribution*

As a first contribution, we provide an analytical power analysis for the Wald, LR, score, and gradient statistic applicable to non-exponential family models and MML estimation. In this context, we formulate the gradient statistic in the context of testing linear hypotheses, which is a second contribution. We thereby provide power analysis for relevant models and hypothesis tests, e.g., for a test of a Rasch model against a 2PL model or examining DIF in 2PL or GPCM models.

An important obstacle in the application of the proposed analytical power analysis is a strongly increased computational load when larger numbers of items are considered. As a third contribution, we therefore present a sampling-based method. Although it has been already presented in a similar form (Guastadisegni et al., 2021; Gudicha et al., 2017), we apply a different computation with the aim of providing a more accurate result. Lastly, we provide an R package that implements the power analysis for user-defined parameter sets and hypotheses at https://github.com/flxzimmer/irtpwr.

In the following sections, we present the different approaches and evaluate them in a test of a Rasch against a 2PL model, a test for differential item functioning (DIF), and a test of a PCM against a GPCM model in an extensive simulation study. We contrast the power of the computationally simpler gradient test with that of the other, more established tests. Furthermore, the application to real data is showcased in the context of the PISA study. Finally, we discuss some limitations and give an outlook on possible extensions.

## Power Analysis

We will first define a general IRT model for which we assume local independence of items and unidimensional person parameters that are independent and identically distributed. As we explain in the Discussion section, we can obtain similar results if we assume multidimensional person parameters. The probability distribution of the item response function is expressed as $f_{{\varvec{\beta }},\theta _v}({\varvec{x}})$, where ${\varvec{\beta }}$ represents the item parameters and $\theta _v$ denotes the unidimensional person parameter for person $v=1 \ldots n$. The vector ${\varvec{\beta }}$ depends on the specific model, but shall generally have length *l*. For the Rasch model, for example, there is a common slope parameter and an intercept parameter for each item such that *l* is one plus the number of items *I*. This corresponds to the number of identifiable parameters in an MML estimation of the Rasch model. Furthermore, let *X* be a discrete random variable with realizations ${\varvec{x}} \in \{1,\ldots ,K\}^I$. Here, *K* is the number of different response categories and *I* is the number of items. Each possible value ${\varvec{x}}$ is therefore a vector of length *I* that represents one specific pattern of answers across all items.

We consider the test of a linear hypothesis using the example of testing a Rasch model against a 2PL model. The use of such linear hypotheses provides a flexible framework for power analyses. The null hypothesis is expressed as $T({\varvec{\beta }})={\varvec{c}}$ or equivalently

1 $$\begin{aligned} A{\varvec{\beta }} = {\varvec{c}}, \end{aligned}$$

where *T* is a linear transformation of the item parameters, $T: {\mathbb {R}}^l \rightarrow {\mathbb {R}}^m \text { with } m\le l$. Let ${\varvec{c}}\in {\mathbb {R}}^m$ be a vector of constants and *A* the unique matrix with *m* rows and *l* columns that represents *T*. In the following, we will denote a set of item parameters that follow the null hypothesis by ${\varvec{\beta }}_0 \in B_0 = \{{\varvec{\beta }}|A{\varvec{\beta }}={\varvec{c}}\}$. Similarly, we will refer to parameters that follow the alternative as ${\varvec{\beta }}_a \in B_a = \{{\varvec{\beta }}|A{\varvec{\beta }}\ne {\varvec{c}}\}$. To describe the 2PL model for the test of a Rasch against a 2PL model, let the probability of a positive answer to item $i=1\ldots I$ be given by

2 $$\begin{aligned} P_{{\varvec{\beta }},\theta }(x_i=1) = \frac{1}{1+ \exp (-(a_{i} \theta + d_{i}))} \end{aligned}$$

with slope parameter $a_{i}$ and intercept parameter $d_{i}$. The slope parameters of the 2PL model are allowed to differ between the items, while they take on a common value in the Rasch model. We can summarize the slope and intercept parameters of the 2PL model introduced in Eq. 2 in a vector ${\varvec{\beta }} = (a_1,d_1,\ldots , a_I,d_I)$. The Rasch model is nested within the 2PL model and can be obtained if we set all slope parameters to a common value. One way to express the associated linear hypothesis *T* and design matrix *A* is

3 $$\begin{aligned} T({\varvec{\beta }})= & {} A{\varvec{\beta }} = \begin{pmatrix} a_1 - a_2 \\ \vdots \\ a_{i-1} - a_i \\ \vdots \\ a_{I-1} - a_{I} \\ \end{pmatrix} = 0 , \end{aligned}$$

4 $$\begin{aligned} A= & {} \begin{pmatrix} 1&{}0&{}-1&{}0&{}\ldots &{}0&{}0\\ \vdots &{} &{} &{} \ddots &{} &{}&{}\vdots \\ 0&{}\ldots &{}0&{}1&{}0&{}-1&{}0 \\ \end{pmatrix}. \end{aligned}$$

An analytical approach for power analysis is based on the fact that the statistics asymptotically follow a different distribution under the null hypothesis than under an alternative hypothesis. Under the null hypothesis, they asymptotically follow a central $\chi ^2$ distribution (Silvey, 1959; Terrell, 2002; Wald, 1943). Under an alternative hypothesis, they asymptotically follow the same noncentral $\chi ^2$ distribution with $\lambda \ne 0$ (Lemonte & Ferrari, 2012). For a proof of the asymptotic distribution under an alternative, we need to assume that parameters ${\varvec{\beta }}_a$ converge to ${\varvec{\beta }}_0$ for $n \rightarrow \infty$. A similar method was used for CML and exponential family models (Draxler & Alexandrowicz, 2015) and for MML and tests based on contingency tables (Maydeu-Olivares & Montaño, 2013). The asymptotic distribution of the statistics depends on the consistency of the maximum likelihood (ML) parameter estimator (Casella & Berger, 2002). The proof of the consistency itself relies on regularity conditions, such as the identifiability of parameters. Under the null hypothesis and weak regularity conditions, the estimated parameters $\hat{{\varvec{\beta }}}$ converge to the true parameters ${\varvec{\beta }}_0$ with a multivariate normal distribution,

$$\begin{aligned} \hat{{\varvec{\beta }}}\xrightarrow {n\rightarrow \infty }{\varvec{\beta }}_0 \implies \sqrt{n}(\hat{{\varvec{\beta }}}-{\varvec{\beta }}_0) \xrightarrow []{d} N[0,\Sigma _{{\varvec{\beta }}_0}]. \end{aligned}$$

Now, if the estimate $\hat{{\varvec{\beta }}}$ converges to a set of parameters following the alternative, ${\varvec{\beta }}_a$, it follows that

$$\begin{aligned} \hat{{\varvec{\beta }}}\xrightarrow {n\rightarrow \infty }{\varvec{\beta }}_a \implies \left\Vert \sqrt{n}(\hat{{\varvec{\beta }}}-{\varvec{\beta }}_0)\right\Vert \xrightarrow {n\rightarrow \infty } \infty . \end{aligned}$$

This is the case regardless of the specific choice of ${\varvec{\beta }}_a$ and ${\varvec{\beta }}_0$, since $\sqrt{n}(\hat{{\varvec{\beta }}}-{\varvec{\beta }}_0) \xrightarrow {n\rightarrow \infty } \sqrt{n}{\varvec{\delta }}$ with ${\varvec{\delta }} ={\varvec{\beta }}_a-{\varvec{\beta }}_0\ne 0$. Since asymptotic normality does not apply in this case, the statistics will not follow a $\chi ^2$ distribution. Instead, as Wald (1943) noted, the power of the respective tests converges to 1 for $n\rightarrow \infty$. Therefore, to derive an asymptotic distribution under the alternative, we need to consider a deviation that shrinks with the sample size. We set

5 $$\begin{aligned} {\varvec{\beta }}_n = {\varvec{\beta }}_0 + \frac{{\varvec{\delta }}_n}{\sqrt{n}} \end{aligned}$$

and assume ${\varvec{\delta }}_n$ is chosen in a way that ${\varvec{\beta }}_n$ follows the alternative hypothesis for all $n\in {\mathbb {N}}$. In this case,

$$\begin{aligned} \hat{{\varvec{\beta }}}\xrightarrow {n\rightarrow \infty }{\varvec{\beta }}_n \implies \sqrt{n}(\hat{{\varvec{\beta }}}-{\varvec{\beta }}_0) \xrightarrow []{d} N[{\varvec{\delta }}_n,\Sigma _{{\varvec{\beta }}_n}], \end{aligned}$$

from which we can conclude the asymptotic noncentral $\chi ^2$ distribution. Moreover, Silvey (1959) notes the asymptotic equality of the Wald, LR, and score statistics for this scenario.

The procedure to apply Eq. 5 to finite samples is as follows. Given an alternative ${\varvec{\beta }}_a$, a null parameter set ${\varvec{\beta }}_r$ as described in the sequel in Eq. 10, and a sample size *n*, we simply set ${\varvec{\delta }}_n = \sqrt{n}({\varvec{\beta }}_a-{\varvec{\beta }}_r)$. Then, ${\varvec{\beta }}_n = {\varvec{\beta }}_a$. With ${\varvec{\beta }}_n$ defined this way, the proposed distribution will hold asymptotically, and the error for the finite sample case may be investigated for practical relevance.

By application of these technical details, the noncentrality parameters $\lambda$ are obtained by evaluating the statistics at the population parameters (Silvey, 1959). Specifically,

6 $$\begin{aligned} \lambda ({\varvec{\beta }},n)= S({\varvec{\beta }},n) \end{aligned}$$

where *S* represents the Wald, LR, score, or gradient statistic. The parameter set ${\varvec{\beta }}$ represents the population parameter of the consistent ML estimator $\hat{{\varvec{\beta }}}$. The noncentrality parameters also depend on an assumed person parameter distribution that we specify in Eq. 8, but omit from the notation. As outlined above, in case ${\varvec{\beta }}$ follows an alternative, ${\varvec{\beta }} \in B_a$, the noncentrality parameters in Eq. 6 accurately describe the respective distributions for $n \rightarrow \infty$ and ${\varvec{\beta }}$ converging to ${\varvec{\beta }}_0$. We may also calculate the noncentrality parameters to approximate the distributions in finite samples and for fixed values of ${\varvec{\beta }}$. As they rely on an asymptotic result, the agreement of the corresponding expected and observed distributions will be higher in larger samples and for lower differences $|{\varvec{\beta }} - {\varvec{\beta }}_0|$, i.e., lower effect sizes. The reliance on these results can be considered common practice according to Agresti (2002) and has been shown to involve only minor errors in a simulation study by Draxler and Alexandrowicz (2015) in the CML context. Also, note that the test of the null hypothesis, i.e., a test against a central $\chi ^2$ distribution, involves a similar assumption.

**Fig. 1:**
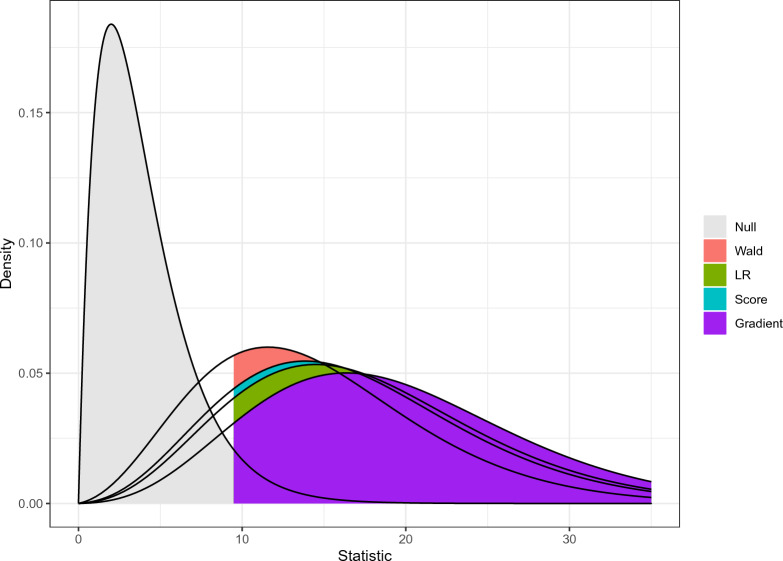
Distributions of the Wald, LR, score, and gradient statistics under the null and an alternative hypothesis in a test of a Rasch versus a 2PL model. The parameters used stem from a model fit of the five items in the “LSAT7” dataset, which is publicly available in the mirt package (Chalmers, 2012). The curve labeled “Null” represents a central $\chi ^2$ distribution that applies to all four statistics under the null hypothesis. For the other four curves, the colored areas under the curve represent the power of the corresponding test under the alternative.

To illustrate the procedure, we again consider testing a Rasch model against a 2PL model. Assuming variable slope parameters, and therefore, a deviation from the Rasch model, all four statistics are expected to behave differently than under the null hypothesis. Using the noncentrality parameters in Eq. 6, we obtain an expected distribution for each of the statistics under the alternative (Fig. 1).

Note that in contrast to the asymptotic case, the noncentrality parameters are not necessarily the same for the four tests. For each of the statistics, the power is represented by the area under the graphs that is above the critical value for rejecting the null hypothesis.

The formula for the noncentrality parameters in Eq. 6 are further explained in Sect. 1.1. For an assessment of the observed and expected distributions, we conduct extensive simulation studies for some practically relevant use cases and common sample sizes in Sect. 2.

### Expected Noncentrality Parameters

Two concepts necessary for the estimation of the expected noncentrality parameters are the expected covariance matrix and the expected restricted parameters, which we will briefly discuss.

#### Expected Covariance Matrix

Let the expected covariance matrix for a parameter set ${\varvec{\beta }}$ in a sample of size *n* be denoted by ${\varvec{\Sigma }}({\varvec{\beta }},n)$. Generally, it is given by the inverse of the information matrix, ${\varvec{\Sigma }}({\varvec{\beta }},n) = {\varvec{I}}^{-1}({\varvec{\beta }},n).$ A range of different methods has been suggested to estimate the information matrix (Yuan et al., 2014). One can distinguish expected and observed information matrices. In general, observed and expected information can lead to different results (Bradlow, 1996) and observed information is preferable, e.g., in the presence of misspecification (Efron & Hinkley, 1978; Yuan et al., 2014). However, observed information matrices require empirical data for their calculation, while expected information matrices can also be calculated in the absence of data and are therefore the only feasible option for a power analysis. The expected Fisher information matrix is given by:

$$\begin{aligned} {\varvec{I}}_{F}({\varvec{\beta }},n)= -n E_{{\varvec{x}}}[\ddot{l}_{{\varvec{\beta }}}({\varvec{x}})], \end{aligned}$$

where

7 $$\begin{aligned} E_{{\varvec{x}}}[\ddot{l}_{{\varvec{\beta }}}({\varvec{x}})] =&\sum _{{\varvec{x}} \in X}\ddot{l}_{{\varvec{\beta }}}({\varvec{x}})g_{{\varvec{\beta }}}({\varvec{x}}), \nonumber \\ \ddot{l}_{{\varvec{\beta }}}({\varvec{x}}) =&\frac{\partial ^2 l_{{\varvec{\beta }}}({\varvec{x}})}{\partial {\varvec{\beta }}^2}, \nonumber \\ l_{{\varvec{\beta }}}({\varvec{x}}) =&\log (g_{{\varvec{\beta }}}({\varvec{x}})), \end{aligned}$$

8 $$\begin{aligned} g_{{\varvec{\beta }}}({\varvec{x}}) =&E_{\theta }[f_{{\varvec{\beta }},\theta }({\varvec{x}})] = \int f_{{\varvec{\beta }},\theta }({\varvec{x}}) \Phi (\theta )\textrm{d}\theta . \end{aligned}$$

Here, *X* is the set of all possible response patterns, and $f_{{\varvec{\beta }},\theta }$ is the probability distribution of the respective IRT model. Instead of $\theta _v$, which refers to the ability parameter of person *v* in an observed dataset, we consider $\theta$ here as a general person parameter of which we take the expectation across an assumed population distribution $\Phi$, e.g., a standard normal distribution. The probability of observing a response pattern ${\varvec{x}}$ given ${\varvec{\beta }}$ is denoted by $g_{{\varvec{\beta }}}({\varvec{x}})$. Finally, the product $n E_{{\varvec{x}}}[\ddot{l}_{{\varvec{\beta }}}({\varvec{x}})]$ uses the independence of observations, i.e., persons are assumed to be drawn randomly from the population of interest. It follows that

9 $$\begin{aligned} {\varvec{\Sigma }}({\varvec{\beta }},n) = \frac{1}{n} {\varvec{\Sigma }}({\varvec{\beta }},1). \end{aligned}$$

The variances of the parameter estimators, which lie on the diagonal of the covariance matrix, decrease accordingly with increasing sample size.

#### Expected Restricted Parameters

Consider the case that the LR statistic is used to differentiate between two models, e.g., a Rasch and a 2PL model. As outlined in Sect. 1.1.4, ML estimation is performed for both models, resulting in two separate estimated parameter sets. In the following, we want to infer some properties of the respective expected parameter sets to inform a power analysis. We refer to the ML parameters of the nested model—here, the Rasch model—as the restricted parameters ${\varvec{\beta }}_r$. Considering the corresponding linear hypothesis, the restricted parameters follow the null hypothesis, ${\varvec{\beta }}_r \in B_0$. Out of all parameters that follow the null hypothesis, ${\varvec{\beta }}_0 \in B_0$, ${\varvec{\beta }}_r$ exhibit the highest likelihood. We may generally define them as

10 $$\begin{aligned} {\varvec{\beta }}_r = \mathop {\text {arg max}}\limits _{{{\varvec{\beta }}}_0} \in B_0 \sum _{{\varvec{x}} \in X} l_{{\varvec{\beta }}_0}({\varvec{x}})g_{{\varvec{\beta }}}({\varvec{x}}) \end{aligned}$$

for an analytical power analysis. Here, *l* is the log probability of a specific response pattern, as defined in Eq. 7. Note that $g_{{\varvec{\beta }}}$ as specified in Eq. 8 depends on the true item parameters ${\varvec{\beta }}$. To find the maximum in Eq. 10, an implementation of the Broyden–Fletcher–Goldfarb–Shanno (BFGS) algorithm in the stats package in R (R Core Team, 2021) was used throughout this paper.

When ${\varvec{\beta }} \in B_a$ follows an alternative hypothesis, the restricted parameters obtained by ML estimation cannot be the data generating model, ${\varvec{\beta }}_r \ne {\varvec{\beta }}$. For the purpose of a power analysis, we assume that a ML estimator $\hat{{\varvec{\beta }}}_r$ converges to a unique ${\varvec{\beta }}_r$ for $n \rightarrow \infty$. In our example, this implies that the ML estimates of the restricted model, which assume a common value for the slope parameters, are unique. As will also be illustrated in our simulation study in Sect. 2, this assumption typically holds.

#### Wald Statistic

We may now derive the noncentrality parameters in Eq. 6 using a similar approach as in Draxler (2010) and Draxler and Alexandrowicz (2015). Let ${\tilde{X}}$ denote an observed dataset and $h_{{\tilde{X}}}({\varvec{x}})$ denote the frequency of a response pattern ${\varvec{x}}$ in the dataset ${\tilde{X}}$. The estimated item parameters $\hat{{\varvec{\beta }}}_r$ are retrieved using a consistent ML estimator.

The Wald statistic $S_1$ is based on the parameter estimates and their covariances (Wald, 1943; see also Glas & Verhelst 1995). The statistic is given by

11 $$\begin{aligned} S_1(\hat{{\varvec{\beta }}},{\tilde{X}}) = (A\hat{{\varvec{\beta }}}-{\varvec{c}})'[A{\varvec{\Sigma }}(\hat{{\varvec{\beta }}},{\tilde{X}})A']^{-1}(A\hat{{\varvec{\beta }}}-{\varvec{c}}), \end{aligned}$$

where ${\varvec{\Sigma }}(\hat{{\varvec{\beta }}},{\tilde{X}})$ is a variance–covariance matrix using the estimated parameters and the observed dataset. If we replace the estimator $\hat{{\varvec{\beta }}}$ with its population parameter ${\varvec{\beta }}$ and consider an expected covariance matrix for a sample of size *n*, we get

$$\begin{aligned} \lambda _{1}({\varvec{\beta }},n)&= S_1({\varvec{\beta }},n) \\&= (A{\varvec{\beta }}-{\varvec{c}})'[A{\varvec{\Sigma }}({\varvec{\beta }},n)A']^{-1}(A{\varvec{\beta }}-{\varvec{c}})\\&= n(A{\varvec{\beta }}-{\varvec{c}})'[A{\varvec{\Sigma }}({\varvec{\beta }},1)A']^{-1}(A{\varvec{\beta }}-{\varvec{c}}), \end{aligned}$$

where ${\varvec{\Sigma }}({\varvec{\beta }},n)$ is an expected variance–covariance matrix as specified in Sect. 1.1.1 and the last equality uses the inverse proportionality of the covariance matrix from Eq. 9. If ${\varvec{\beta }}$ follows the null hypothesis, then $A{\varvec{\beta }}-{\varvec{c}}=0$ and the noncentrality parameter is $\lambda _{1}({\varvec{\beta }},n)=0$ for any sample size n.

#### Likelihood Ratio Statistic

The LR statistic directly compares the likelihoods of a restricted model and an unrestricted model (Silvey, 1959 see also Glas & Verhelst, 1995). The statistic for an observed dataset is given by

$$\begin{aligned} S_2(\hat{{\varvec{\beta }}},\hat{{\varvec{\beta }}}_r,{\tilde{X}}) = 2\sum _{{\varvec{x}} \in {\tilde{X}}} \big (l_{\hat{{\varvec{\beta }}}}({\varvec{x}})-l_{\hat{{\varvec{\beta }}}_r}({\varvec{x}})\big )h_{{\tilde{X}}}({\varvec{x}}) \end{aligned}$$

where *l* is given in Eq. 7. ML estimation is performed for both the unrestricted and the restricted parameter set. Analogous to the Wald statistic, the noncentrality parameter is given as

$$\begin{aligned} \lambda _{2}({\varvec{\beta }},n)&= S_2({\varvec{\beta }},{\varvec{\beta }}_r,n)\\&= 2\sum _{{\varvec{x}} \in X} \big (l_{{\varvec{\beta }}}({\varvec{x}})-l_{{\varvec{\beta }}_r}({\varvec{x}})\big ) n g_{{\varvec{\beta }}}({\varvec{x}})\\&= 2n\sum _{{\varvec{x}} \in X} \big (l_{{\varvec{\beta }}}({\varvec{x}})-l_{{\varvec{\beta }}_r}({\varvec{x}})\big ) g_{{\varvec{\beta }}}({\varvec{x}}), \end{aligned}$$

where ${\varvec{\beta }}_r$ is calculated as in Eq. 10 with respect to a null hypothesis $A{\varvec{\beta }}={\varvec{c}}$ introduced in Eq. 1. Note that the frequency of each response pattern ${\varvec{x}}$ in the population is given by its probability given the true parameters $g_{{\varvec{\beta }}}({\varvec{x}})$ multiplied by the sample size. This uses the assumption of independent and identically distributed person parameters. As we noted above, ${\varvec{\beta }}_r$ is equal to ${\varvec{\beta }}$ when the null hypothesis holds; the noncentrality parameter is then $\lambda _{S_2}({\varvec{\beta }},n)=0$.

#### Score Statistic

The concept of score statistics is to estimate only the restricted parameter set and to consider the gradient of its likelihood function (Rao, 1948 see also Glas & Verhelst, 1995). When the absolute gradient at the restricted parameters is high, we may conclude a bad model fit and discard the null hypothesis. It is based on the assumption that the gradient of the likelihood is close to zero at the true parameter values. The score statistic is given by

12 $$\begin{aligned} S_3(\hat{{\varvec{\beta }}}_r,{\tilde{X}}) = \Big (\sum _{{\varvec{x}} \in {\tilde{X}}}{\dot{l}}_{\hat{{\varvec{\beta }}}_r}({\varvec{x}})h_{{\tilde{X}}}({\varvec{x}})\Big )'{\varvec{\Sigma }}(\hat{{\varvec{\beta }}}_r,{\tilde{X}})\Big (\sum _{{\varvec{x}} \in {\tilde{X}}}{\dot{l}}_{\hat{{\varvec{\beta }}}_r}({\varvec{x}})h_{{\tilde{X}}}({\varvec{x}})\Big ) \end{aligned}$$

for an observed dataset, where ${\dot{l}}$ is the first derivative of the likelihood, Eq. 7. For the noncentrality parameter, we arrive at

13 $$\begin{aligned} \begin{aligned} \lambda _{3}({\varvec{\beta }},n)&= S_3({\varvec{\beta }},n)\\ {}&= \Big (\sum _{{\varvec{x}} \in X}{\dot{l}}_{{\varvec{\beta }}_r}({\varvec{x}}) n g_{{\varvec{\beta }}}({\varvec{x}})\Big )'{\varvec{\Sigma }}({\varvec{\beta }}_r,n)\Big (\sum _{{\varvec{x}} \in X}{\dot{l}}_{{\varvec{\beta }}_r}({\varvec{x}})n g_{{\varvec{\beta }}}({\varvec{x}})\Big ) \\&= n\Big (\sum _{{\varvec{x}} \in X}{\dot{l}}_{{\varvec{\beta }}_r}({\varvec{x}}) g_{{\varvec{\beta }}}({\varvec{x}})\Big )'{\varvec{\Sigma }}({\varvec{\beta }}_r,1)\Big (\sum _{{\varvec{x}} \in X}{\dot{l}}_{{\varvec{\beta }}_r}({\varvec{x}}) g_{{\varvec{\beta }}}({\varvec{x}})\Big ), \end{aligned} \end{aligned}$$

where we can immediately confirm that $\lambda _{S_3}({\varvec{\beta }},n) = 0$ under the null hypothesis.

#### Gradient Statistic

The gradient statistic combines the Wald and score statistics to eliminate the need to calculate an information matrix. It was formulated by Terrell (2002) in the case that *A* is the identity matrix. Subsequently, it was extended to composite hypotheses, where each row of the *A* matrix contains one nonzero entry (Lemonte, 2016). We propose a generalization to arbitrary linear hypotheses. First, consider an alternative version of the Wald statistic in Eq. 11 that uses a covariance matrix ${\varvec{\Sigma }}(\hat{{\varvec{\beta }}_r},{\tilde{X}})$ that is evaluated at the restricted estimates rather than at the unrestricted estimates of the parameters. We can express it as a vector product

14 $$\begin{aligned} S_1^*(\hat{{\varvec{\beta }}},{\tilde{X}}) = [B(A\hat{{\varvec{\beta }}}-{\varvec{c}})]'B(A\hat{{\varvec{\beta }}}-{\varvec{c}}) \end{aligned}$$

where *B* is a solution for $B'B = [A{\varvec{\Sigma }}(\hat{{\varvec{\beta }}},{\tilde{X}})A']^{-1}$. Secondly, consider an equivalent expression of the score statistic in Eq. 12,

15 $$\begin{aligned} S_3^*(\hat{{\varvec{\beta }}}_r,{\tilde{X}}) = {\varvec{k}}'[A{\varvec{\Sigma }}(\hat{{\varvec{\beta }}},{\tilde{X}})A']{\varvec{k}} = {\varvec{k}}'B^{-1}(B^{-1})'{\varvec{k}} \end{aligned}$$

where ${\varvec{k}}$ is a vector of Lagrange multipliers (Silvey, 1959). It is the solution of

16 $$\begin{aligned} \sum _{{\varvec{x}} \in {\tilde{X}}}{\dot{l}}_{\hat{{\varvec{\beta }}}_r}({\varvec{x}})h_{{\tilde{X}}}({\varvec{x}})=-A{\varvec{k}}. \end{aligned}$$

The gradient statistic is then defined as the product of the square roots of the alternative expressions in Eqs. 14 and 15:

17 $$\begin{aligned} S_4(\hat{{\varvec{\beta }}}_r,{\tilde{X}}) = {\varvec{k}}'B^{-1}B(A\hat{{\varvec{\beta }}}-{\varvec{c}}) = {\varvec{k}}'(A\hat{{\varvec{\beta }}}-{\varvec{c}}). \end{aligned}$$

By replacing the frequency $h_{{\hat{X}}}({\varvec{x}})$ by its expected value $n g_\beta ({\varvec{x}})$ in the derivation of the Lagrange multiplier in Eq. 16, we obtain

$$\begin{aligned} \lambda _{4}({\varvec{\beta }},n) = n {\varvec{k}}'(A{\varvec{\beta }}-{\varvec{c}}) \end{aligned}$$

as the noncentrality parameter.

### Sampling-Based Noncentrality Parameters

In the above sections, we presented an analytical method for determining power in the Wald, LR, score, and gradient tests that is applicable to a general class of IRT models outside of the exponential family. The necessary computations quickly become prohibitive for a higher number of items *I* since calculations over all unique response patterns are required. One example is the term

$$\begin{aligned} \sum _{{\varvec{x}} \in X}{\dot{l}}_{{\varvec{\beta }}_r}({\varvec{x}}) g_{{\varvec{\beta }}}({\varvec{x}}) \end{aligned}$$

in the score statistic, Eq. 13, where we need to sum overall $K^I$ possible response patterns in *X*. A questionnaire on a five-point Likert scale and 100 items implies calculations for each of $\sim 7.89\times 10^{69}$ unique patterns, which is infeasible even for modern computers.

For this scenario, we propose a sampling-based approach to approximate the noncentrality parameters in Eq. 6. Similar approaches were presented by Gudicha et al. (2017) and Guastadisegni et al. (2021). It builds upon the assumption of asymptotically $\chi ^2$-distributed statistics, the proportionality of the noncentrality parameter with the sample size, $\lambda ({\varvec{\beta }},n) = n \lambda ({\varvec{\beta }},1)$, as well as the asymptotic convergence of the ML estimators. Given the hypothesized population parameters of the model under the alternative hypothesis, ${\varvec{\beta }}$, and a sample of size *n*, the steps to calculate $\lambda ({\varvec{\beta }},1)$ are:

1. Generate an artificial dataset ${\tilde{X}}$ of size *n* using the item response function $f_{{\varvec{\beta }},\theta }$ and a person parameter distribution $\Phi$.
2. Perform ML estimation and calculate the desired statistics.
3. Estimate the noncentrality parameter $\lambda ({\varvec{\beta }},n)$ by taking the value of the statistics minus the degrees of freedom of the hypothesis test. We can obtain $\lambda ({\varvec{\beta }},1)$ by taking $\lambda ({\varvec{\beta }},n)/n$.

The sample size *n* can be chosen freely according to the available computational resources. With increasing *n*, the estimated noncentrality parameters converge to the noncentrality parameters calculated using the analytical method. As for the analytical method, the resulting noncentrality parameters can be used to approximate the power curve at arbitrary sample sizes.

## Evaluation

The outlined simulation study was designed to test (a) whether the distributions of the test statistics are accurately described by the proposed noncentral $\chi ^2$ distributions and (b) whether the observed power of the statistics is consistent with the respective predictions. The simulation conditions differed in the type of postulated hypothesis, the number of items, the postulated effect size, and the sample size. As a result, we compared the observed and expected statistics with regards to their distribution and power. The respective agreement of expected and observed distribution and hit rate under the null hypothesis was considered as a benchmark. With this design, we aimed to provide empirical evidence for the approximation of the statistics by noncentral $\chi ^2$ distributions given practically relevant effect and sample sizes. We provide the complete code for all reported analyses as online supplemental material.

### Design

We performed a simulation study with 180 conditions by fully crossing three different types of hypotheses, four numbers of items, five sample sizes, and three effect sizes. Under each condition, 5000 artificial datasets were generated.

*Type of hypothesis* The types of the postulated hypotheses were the test of a Rasch model against a 2PL model, a test for DIF in the 2PL model, and a test of a PCM against a GPCM model that we each briefly introduce below.

*Rasch against 2PL* One property of the Rasch model is that items exhibit equal slope parameters. If this is not the case, a 2PL model will generally provide a better fit for the data. A test of the null hypothesis of equal slope parameters is therefore relevant to possibly discard a wrongly assumed Rasch model.

We considered the 2PL model introduced in Eq. 2 and the linear hypothesis of equal slope parameters expressed in Eq. 3 with the design matrix *A* given in Eq. 4. Note that there are many different formulations of the design matrix that imply identical restricted and unrestricted models. All equivalent matrices are given by $B=CA$ with an invertible matrix *C*. As can be seen from Eqs. 11 and 17, the Wald and gradient statistics remain unchanged for all such choices of *C*. Since the implied restricted and unrestricted models are identical, the LR and score statistic are also not affected by the specific choice of *C*. The degrees of freedom of the associated expected noncentral distribution are equal to the number of rows in *A*, i.e., $I-1$ (Glas & Verhelst, 1995).

*DIF* We tested for DIF in one item between two groups, *A* and *B*. If DIF is present, the response function differs between the two groups. For items affected by DIF, test takers with the same abilities might have different solution probabilities. This can be caused by differences in the ${\varvec{d}}$ as well as in the ${\varvec{a}}$ parameters. Consider the null hypothesis that the parameters of the first item are equal in both groups,

$$\begin{aligned} (a_{1A},d_{1A})' =(a_{1B},d_{1B})'. \end{aligned}$$

Therefore, let ${\varvec{\beta }}$ be structured so that the parameters of the first item are replaced by $(a_{1A},d_{1A},a_{1B},d_{1B})'$. The remaining items were estimated jointly for both groups and thereby served as an anchor for the comparison of the item difficulties in the first item. The linear hypothesis *T* and design matrix *A* took the form

$$\begin{aligned} T({\varvec{\beta }})= & {} \begin{pmatrix} a_{1A}-a_{1B} \\ d_{1A}-d_{1B} \end{pmatrix} = 0, \\ A= & {} \begin{pmatrix} 1&{}0&{}-1&{}0&{}0&{}\ldots &{}0\\ 0&{}1&{}0&{}-1&{}0&{}\ldots &{}0\\ \end{pmatrix}. \end{aligned}$$

Note that, as described above, there are many equivalent ways to set up the matrix *A*. The corresponding expected noncentral $\chi ^2$ distribution has two degrees of freedom, i.e., the number of rows in *A*. For simplicity of presentation, a standard normal distribution of the person parameter was assumed in each group. If this assumption cannot be justified, one can, e.g., estimate the mean of the person parameter distribution in each group and add corresponding rows and columns to the design matrix. Also, one may choose other anchoring strategies and, for example, estimate the remaining item parameters separately in each group.

*PCM against GPCM* Testing a PCM against a GPCM for polytomous items is analogous to testing a Rasch model against a 2PL model for binary items. To model polytomous items, PCM and GPCM models feature multiple intercept parameters per item instead of one. If we consider, for example, $K=3$ possible response categories, there are two intercept parameters and, for the GPCM, one slope parameter per item. The probability of answering an item with category $k = 1,2,3$ is for the GPCM (Chalmers, 2012):

$$\begin{aligned} P_{{\varvec{\beta }},\theta }(x_i=k) = \frac{\exp \big ((k-1) a_i \theta + d_{ik}\big )}{\sum _{k=1}^K \exp \big ((k-1) a_i \theta + d_{ik}\big )}. \end{aligned}$$

For identification of the model, we set $d_{i1}=0$. To obtain a PCM in this representation, we set all slope parameters $a_i$ to the same value. The hypothesis was therefore identical to the one for the Rasch against 2PL model, Eq. 3, and the design matrix was set up analogous to Eq. 4.

*Number of items and sample sizes* Four different numbers of items were used, 5, 10, 20, and 50. The sample sizes were 100, 250, 500, 1000, and 3000.

*Effect Sizes* Three different effect sizes labeled “no,” “small,” and “large” were used. The ${\varvec{d}}$ parameters were drawn from a standard normal distribution for both types of hypotheses and all effect sizes. For the Rasch against 2PL hypothesis, the ${\varvec{a}}$ parameters were drawn from a lognormal distribution with mean 1 and standard deviations of 0.22, 0.17, 0.12, 0.10 for the large and 0.16, 0.12, 0.08, 0.05 for the small effect size condition for 5, 10, 20, and 50 items, respectively. The standard deviation of the ${\varvec{a}}$ parameters was chosen smaller for the 50 item condition than for the 10 item condition because this type of deviation from the Rasch model is more easily detected using larger item sets, and we wanted to assure a broad spectrum of resulting power values. For the DIF hypothesis and the small effect size, the item parameters for the first item were set to $a_{1A}=1.125,d_{1A}=0.125$ in the first group and to $a_{1B}=0.875,d_{1B}=-0.125$ in the second group. The respective group differences in both parameters are increased from 0.25 to 0.5 for the large effect size. The ${\varvec{a}}$ parameters for the remaining items were drawn from a lognormal distribution with mean 1 and a standard deviation of 0.1. For the PCM against GPCM hypothesis, the ${\varvec{a}}$ parameters were drawn analogous to the Rasch against 2PL hypothesis, with slightly different numerical values for the standard deviations of the lognormal distribution.

*Estimation of statistics and noncentrality parameters* All analyses were performed with R (R Core Team, 2021). The package mirt (Version 1.34, Chalmers, 2012) was used to fit the IRT models to the artificial datasets. The maximum number of cycles used in the expectation maximization algorithm (Bock & Aitkin, 1981 was set to 5000.

For 5 and 10 items, both the analytical and sampling-based power analysis approach were used while for larger numbers of items (20 and 50), only the sampling-based approach is feasible for computational reasons outlined in Sect. 1.2. In the sampling-based approach, the freely selectable sample size for the artificial dataset ${\tilde{X}}$ was set to 1,000,000 for the former and to 100,000 for the latter conditions. Analogously, calculation of the expected Fisher matrix used in the observed Wald and score statistics is only feasible for the conditions with lower numbers of items. For the conditions with higher numbers of items, we employed a sampling-based approach to approximate the expected Fisher matrix. We therefore first generated a larger artificial dataset and then, using a model fit constrained to the same item parameters, calculated an observed information matrix. Since it is the default option in mirt, the method described by Oakes (1999) was used for calculating the observed information (Chalmers, 2018). It can be calculated quickly and converges toward the expected Fisher information matrix in large samples. It was also applied in the sampling-based power analysis approach. The sample size used for the artificial dataset for approximating the expected Fisher matrix was 100,000 for lower and 10,000 for higher item numbers. For the observed statistics in the sampling-based power analysis approach, we increased these numbers by a factor of 10 since they accounted for only a small portion of the total computation time.

*Analysis methods* For each artificial dataset, the respective hypothesis was tested by fitting the implied IRT models and calculating the statistics. QQ-plots were used for visual evaluation of the resulting distributions of the statistics. For the plots displaying the observed and expected hit rate, a 99% confidence envelope was plotted as a visual anchor (Fox, 2016). The width of the confidence envelope was calculated according to

$$\begin{aligned} 2.576\frac{\sqrt{n_{r}\cdot p_e \cdot (1-p_e)}}{n_r}, \end{aligned}$$

where $n_r$ denotes the number of simulation runs and the expected hit rate $p_e$.

### Results

#### Agreement of the Distributions

**Fig. 2:**
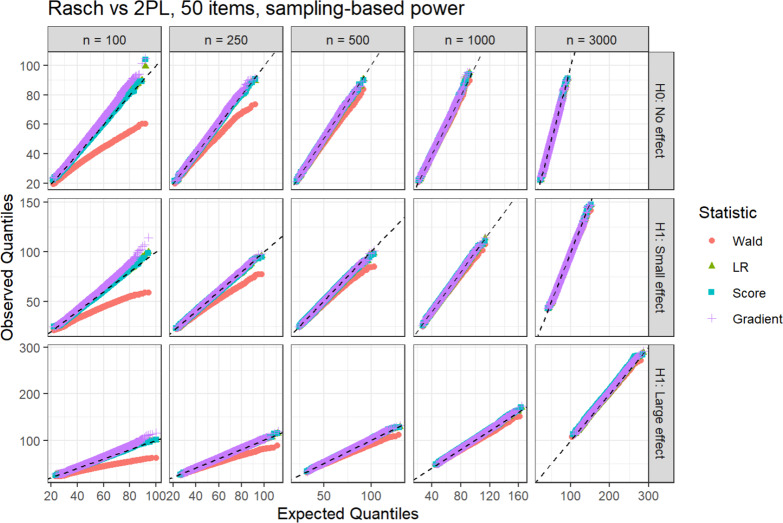
QQ-plots for the Rasch versus 2PL hypothesis with 50 items and the sampling-based power analysis method.

For illustration of the agreement of expected and observed distributions, we want to highlight the QQ-plots for two conditions. The QQ-plots for all other conditions are included in Appendix A. For 50 items and the Rasch versus 2PL hypothesis (Fig. 2), some deviations between the expected and observed distributions are visible for the Wald statistic.

**Fig. 3:**
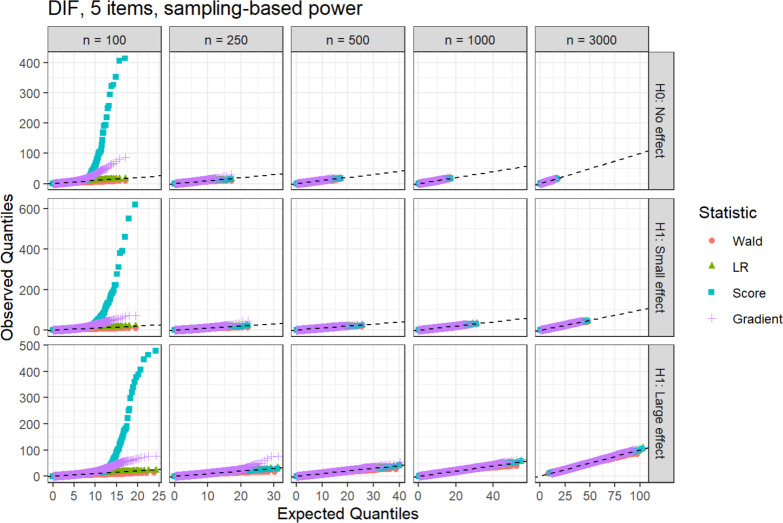
QQ-plots for the DIF hypothesis with 5 items and the sampling-based power analysis method.

Especially for sample sizes 100 and 250, the observed Wald statistic takes on overall smaller values than expected, but the agreement visibly increases with higher sample sizes. For 5 items and the DIF hypothesis (Fig. 3), large deviations between the expected and observed distributions are visible for the gradient and score statistic, mainly for sample sizes 100 and 250.

The statistics take on larger values than expected, and the agreement again increases with higher sample sizes.

Across all other conditions, a similar pattern can be seen. The LR statistic shows a good agreement under all conditions. The score and gradient statistics are larger than expected for sample sizes 100 and 250, and exhibit an increasing agreement for the larger sample sizes. The Wald statistic tends to lie below the respective expectation, with a decreasing severity for higher sample sizes and smaller effect sizes. The sampling-based approach displays an equal fit as the analytical method in the conditions with 5 and 10 items (Figs. 3, 8, 9, 10, 11, 13, 14, 15, 18, 19, 20, 21).

#### Agreement of Power

Since the results of the analytical and sampling-based power analysis were similar and the latter covered all conditions studied, we will first present the results for the sampling-based approach and then discuss the differences between the two. We nevertheless included analogous plots for the analytical approach in Appendix B.

**Fig. 4:**
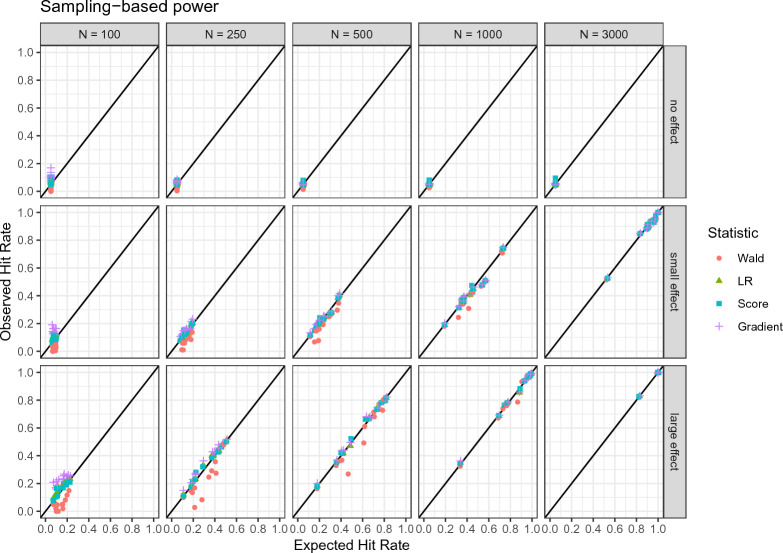
Observed and expected hit rates by effect size and sample size using the sampling-based power analysis approach.

Figure 4 visualizes the expected and observed hit rate ordered by effect size and sample size.

The tables in Appendix C display the results in more detail for each condition. It can be seen that the expected and observed hit rates are largely in agreement under most conditions. Most observed values are within a 99% confidence envelope, which is the expected variability given a perfect agreement taking into account the number of simulations. Given an effect is present, the power increases with a higher effect size and a larger sample size. We can observe that the hit rate for data generated under the null hypothesis (“no effect”) approaches the nominal value of .05 with larger sample sizes.

**Fig. 5:**
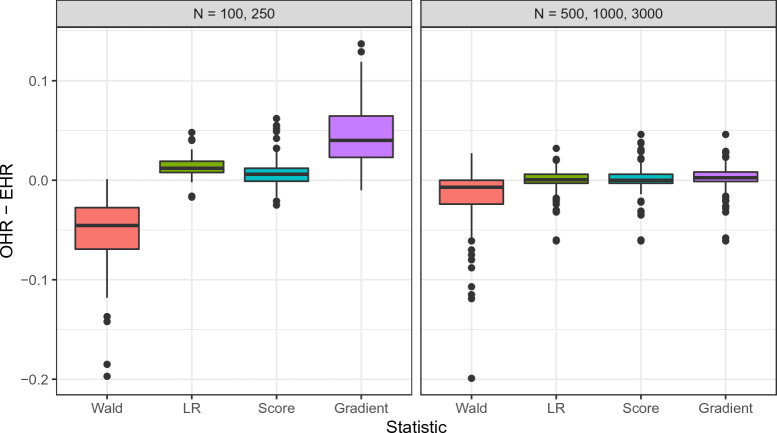
OHR: Observed hit rate. EHR: Expected hit rate. The expected hit rate was calculated using the sampling-based power analysis approach.

Figure 5 summarizes the differences of observed and expected hit rate for lower and higher sample sizes.

In particular for conditions with smaller sample sizes, the power for the Wald statistic is in many cases lower than the respective expectation. This is largely consistent with the observations on the agreement of the distributions. For lower sample sizes, we can also notice that the gradient statistic has a higher hit rate than expected, which again reflects the distributions of the statistic in these scenarios. The differences of observed and expected hit rate become notably lower for larger sample sizes, with only the Wald statistic exhibiting some higher deviations.

**Fig. 6:**
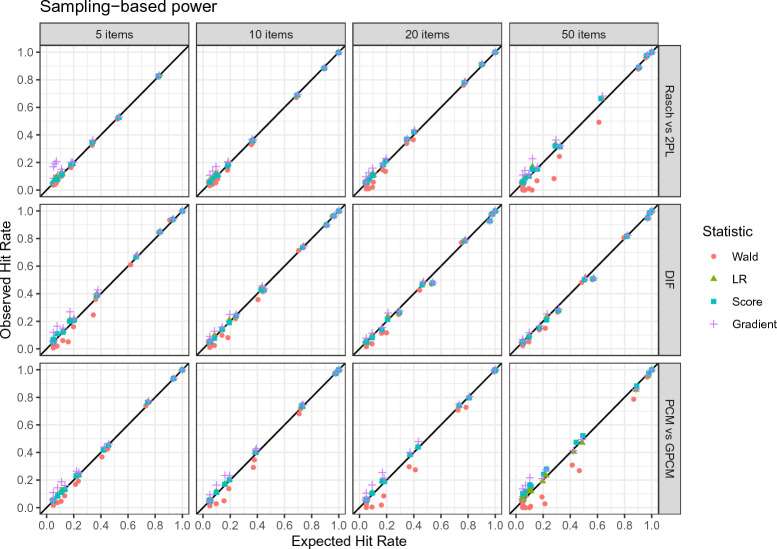
Observed and expected hit rates by hypothesis type and the number of items using the sampling-based power analysis approach.

Figure 6 provides a visualization of the hit rates ordered by the hypothesis type and the number of items.

We find that the agreement is higher for lower numbers of items and that it is generally better for the DIF hypothesis.

For the analytical power analysis, which was calculated for conditions with 5 or 10 items, we observed only small differences to the sampling-based approach. The mean difference to the power calculated from the sampling-based approach in all relevant conditions (only small or large effect sizes) is $1.167 \times 10^{-4}$ and its standard deviation is .007. The mean absolute difference of the power rates between both approaches is .004 and the maximum absolute difference we observed is .021.

## Real Data Application

In the 2015 run of the PISA study, the item responses were modeled with the 2PL and GPCM. This was an important change to the 2012 run, where the more restrictive Rasch and partial credit models were used (OECD, 2017). This motivates the question: “If the PISA data can be described by the 2PL model, what sample size is minimally needed to discard the simpler Rasch model”? As an illustration, we regarded all dichotomous items in two clusters of mathematics items, M1 and M2. The parameters converted to the slope/intercept parametrization are listed in Table 1 (OECD, 2017).

**Table 1:** Parameters for the M1 and M2 PISA 2015 items

Item	M1		M2	
	a	d	a	d
1	1.00	0.96	0.62	$-$0.09
2	1.00	0.67	1.00	0.02
3	1.00	0.67	1.00	0.61
4	1.00	0.28	0.59	0.91
5	1.43	0.11	1.78	$-$0.91
6	0.69	$-$0.04	2.30	$-$1.80
7	1.70	$-$0.87	0.76	$-$0.25
8	1.48	$-$0.65	1.00	$-$0.03
9	1.00	$-$0.25	0.63	0.03
10			1.00	0.09

In the original analysis, the 2PL model was only used for items that showed poor fit to the Rasch model, and therefore some slope parameters are exactly 1.

**Fig. 7:**
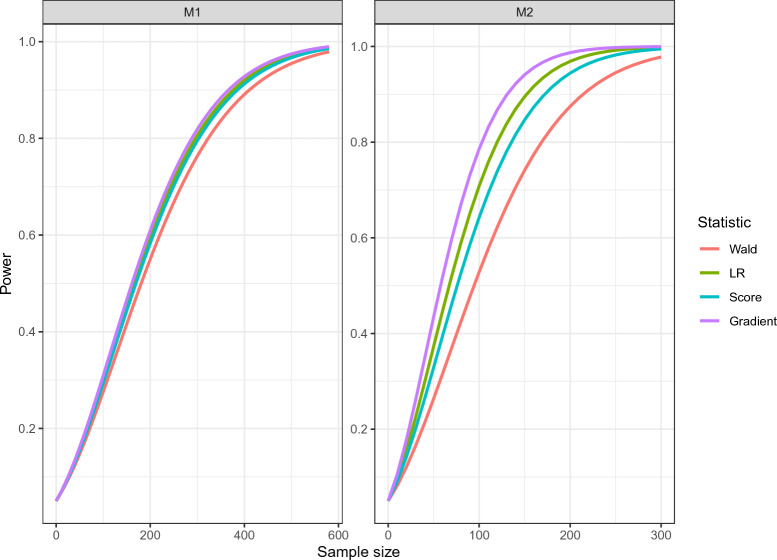
Power curves for testing a Rasch against a 2PL model for two PISA item clusters.

**Fig. 8:**
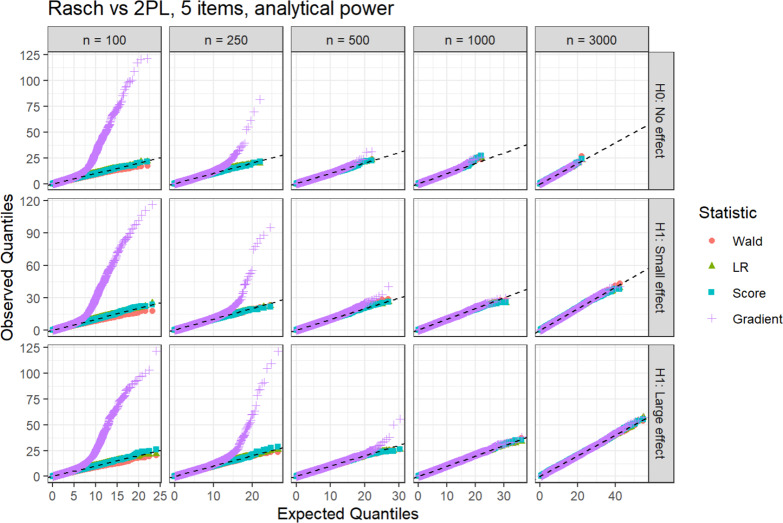
QQ-plots for the Rasch versus 2PL hypothesis with 5 items and the analytical power analysis method.

**Fig. 9:**
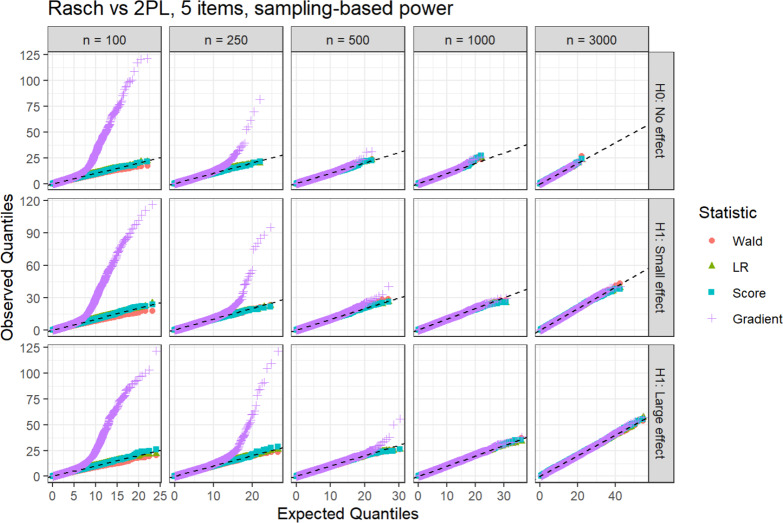
QQ-plots for the Rasch versus 2PL hypothesis with 5 items and the sampling-based power analysis method.

**Fig. 10:**
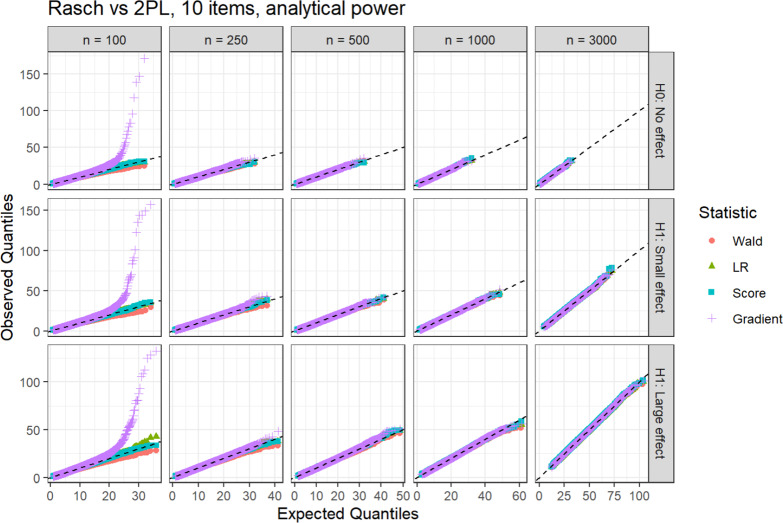
QQ-plots for the Rasch versus 2PL hypothesis with 10 items and the analytical power analysis method.

**Fig. 11:**
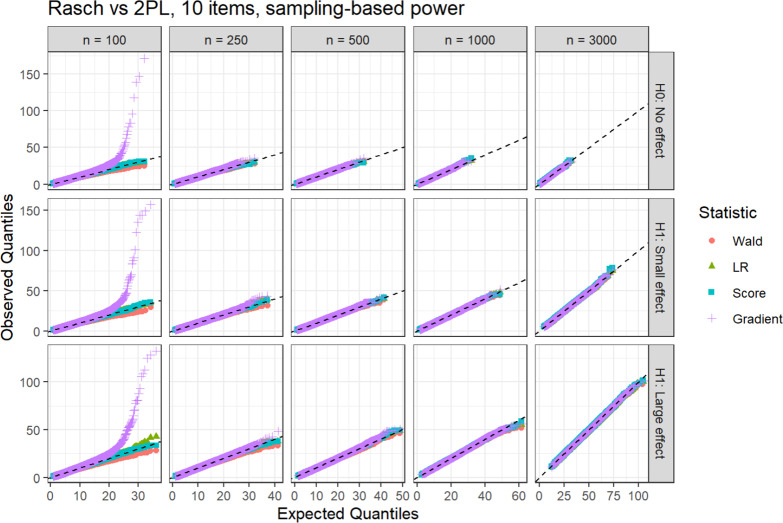
QQ-plots for the Rasch versus 2PL hypothesis with 10 items and the sampling-based power analysis method.

**Fig. 13:**
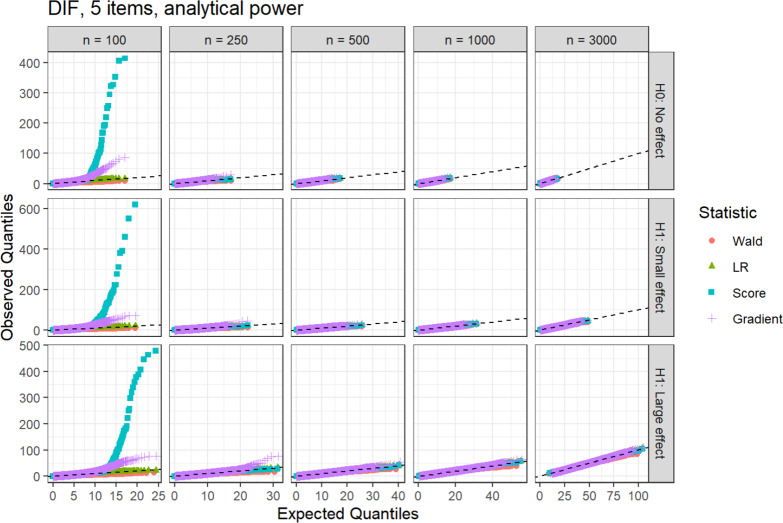
QQ-plots for the DIF hypothesis with 5 items and the analytical power analysis method.

**Fig. 14:**
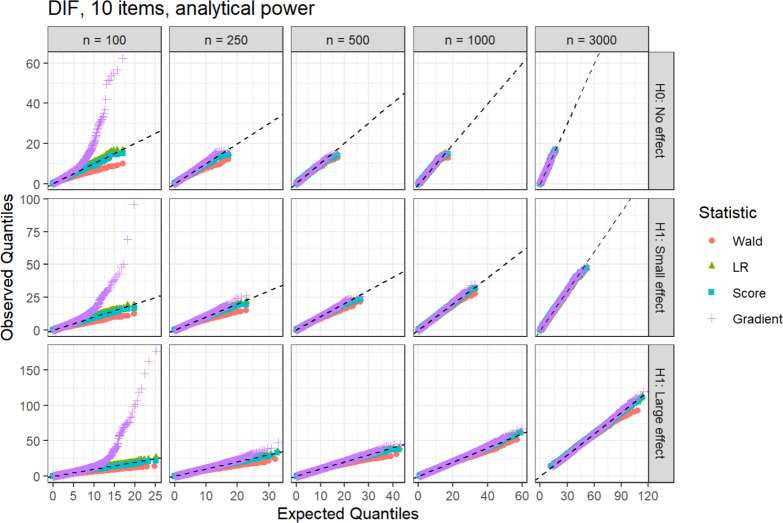
QQ-plots for the DIF hypothesis with 10 items and the analytical power analysis method.

**Fig. 15:**
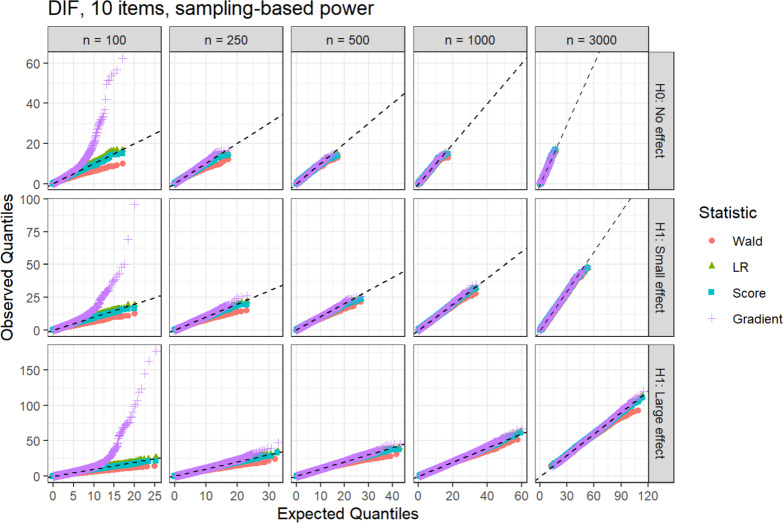
QQ-plots for the DIF hypothesis with 10 items and the sampling-based power analysis method.

**Fig. 18:**
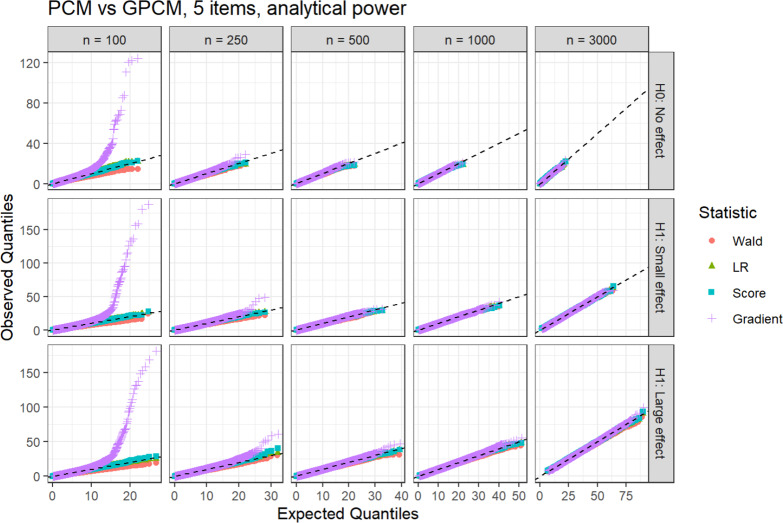
QQ-plots for the PCM versus GPCM hypothesis with 5 items and the analytical power analysis method. To maintain readability, three outliers with values greater than 1000 were removed for the Wald statistic in the condition using N = 100 and the large effect size.

**Fig. 19:**
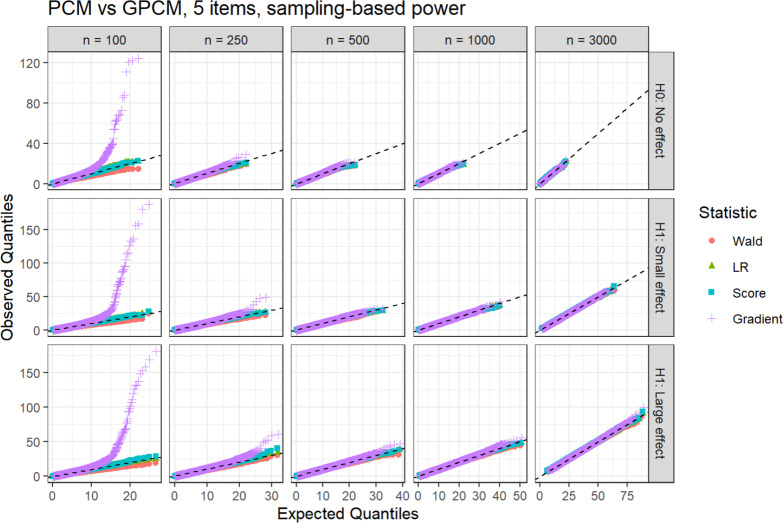
QQ-plots for the PCM versus GPCM hypothesis with 5 items and the sampling-based power analysis method. To maintain readability, three outliers with values greater than 1000 were removed for the Wald statistic in the condition using $N = 100$ and the large effect size.

**Fig. 20:**
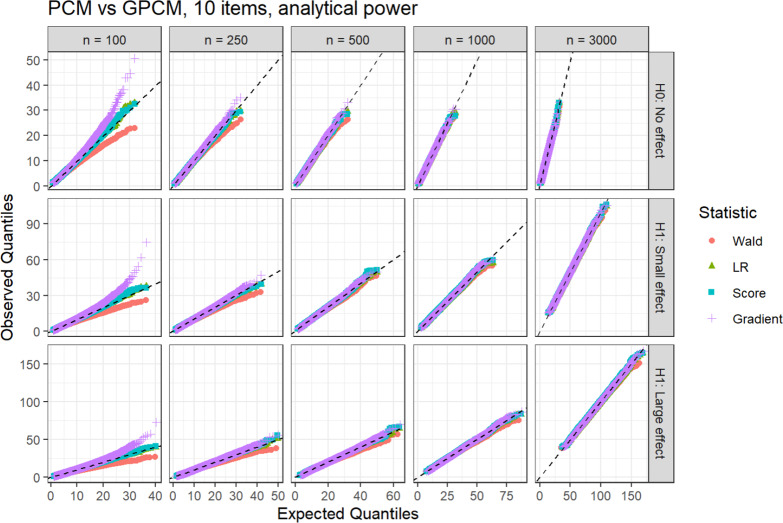
QQ-plots for the PCM versus GPCM hypothesis with 10 items and the analytical power analysis method.

**Fig. 21:**
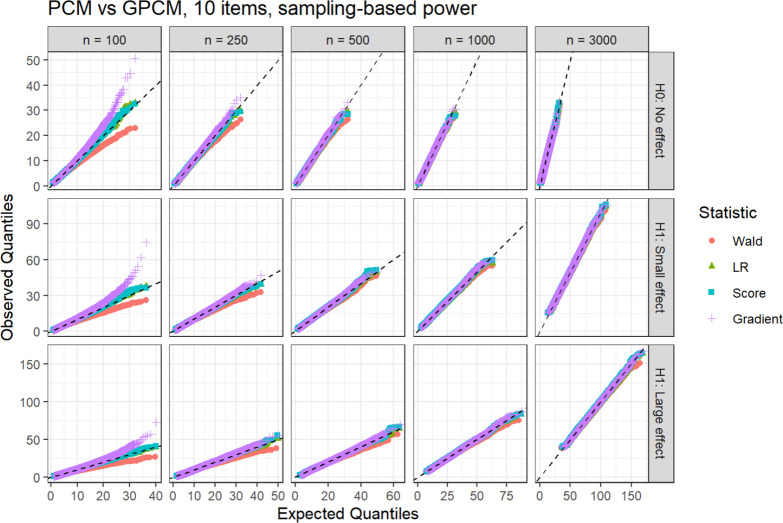
QQ-plots for the PCM versus GPCM hypothesis with 10 items and the sampling-based power analysis method.

For the power analysis, we used the Rasch model as the null model and a 2PL model with the above parameters as the alternative model. We assumed standard normally distributed person parameters and applied a Rasch versus 2PL hypothesis test as described in Sect. 2.1. The relationship of power and sample size using the analytical method is displayed in Fig. 7.

To achieve a power of .9, the resulting sample sizes for the Wald, LR, score, and gradient statistics were 422, 389, 397, and 378, respectively, for the M1 cluster and 233, 166, 188, and 143, respectively, for the M2 cluster. The results underscore the differences among the statistics with the gradient statistic offering the highest power. This illustrates that, under some conditions, small samples may suffice for comparing two IRT models, and much larger samples may be needed under other conditions. Furthermore, since the four statistics are only asymptotically equivalent (Lemonte, 2012; Silvey, 1959; Wald, 1943) one may infer which of the tests has the highest power in a specific scenario.

To further illustrate the dependency of the required sample size on the tested hypothesis, we offer a second example based on the same application. This second application is a DIF test, which plays an important role in educational testing (American Educational Research Association et al., 2014). We can use a power analysis to estimate how likely we will detect actually existing DIF for groups differing in gender, language, age, etc. As an example application using the 2015 PISA data, we assessed the power of detecting DIF for the first item in the M1 cluster. As an alternative hypothesis, we considered two groups, one for which the parameters in Table 1 hold, and another for which the first item was more difficult and had a higher slope ($a = 1$, $d = 0.96$ in the first and $a = 1.2$, $d = 0.5$ in the second group). We assumed standard normally distributed person parameters for all participants. The results indicate that, depending on the statistic, an overall sample size between 1113 and 1140 is required to detect the group difference with a power of .9.

## Discussion

In this paper, we proposed the gradient statistic for arbitrary linear hypotheses and provided two approaches for power analysis for the Wald, LR, score, and gradient statistic applicable to non-exponential family models and MML estimation. In particular, we introduced an analytical as well as a sampling-based approach to approximate the distribution of the statistics under alternative hypotheses.

In an extensive simulation study involving three relevant hypotheses and MML estimation, we demonstrated that the generated approximations suffice for practical purposes and are suitable for application in power estimation and sample size planning. For lower sample sizes, especially $N = 100$, we found some larger deviations in distribution and power, mainly for the Wald, score, and gradient statistics. According to the theoretical results, the statistics show increasing agreement with the proposed distributions asymptotically, i.e., with higher sample sizes. Correspondingly, we observed that the resulting power estimates become more accurate with larger sample sizes. For our medium-sized samples ($N = 500$), the Wald statistic still shows some tendency to be lower than expected, while the other three statistics already exhibit a higher accuracy. Across the studied conditions, the gradient statistic showed the highest expected power, followed by the LR, score, and Wald statistics.

### Limitations

Computational limits of the presented analytical method are reached quickly when large item sets are considered. The required calculation steps grow by $2^I$ since they imply going over all possible patterns for all studied statistics; therefore, for larger item sets, only the sampling-based approach is feasible. We did not find practically relevant differences between the analytical and sampling-based approach in our simulation study. Since the sampling-based approach can be expected to converge to the analytical approach with increasing computational resources, we recommend using the analytical method as long as it is feasible with respect to the number of items. To facilitate the decision on the approach to be used, we provide a function to estimate the computation time of the analytical approach in the R package.

The general line of argumentation in this paper involves the application of asymptotic mathematical results to finite samples. It must be expected that the results are more prone to error with smaller sample sizes and larger effect sizes; however, we note that the dependence on the sample size also applies to the statistics under a true null hypothesis. To avoid this problem, one can use simulation-based approaches (Wilson et al., 2020). The main disadvantage of these are that for sample size planning, in particular, a high computational load can be expected to approximate the relationship between sample size and power. Therefore, in practice, assuming that usually sufficiently large sample sizes and mild deviations from the null hypothesis are already considered relevant, the approaches presented herein might be preferable.

In the instance of a Rasch model as the null hypothesis, one might—besides the presence of DIF or varying item slopes—also consider the influence of guessing. The 3PL model extends the 2PL model by a guessing parameter that lies in the range of 0–1, where 0 represents the absence of guessing and the sufficiency of a 2PL model. When a 3PL model is fit to data generated by a Rasch model, one can expect the estimates for the guessing parameters to deviate from 0 since they lie on the boundary of the parameter space (Brown et al., 2015). Consequently, the Wald, LR, and gradient statistics deviate from a central $\chi ^2$ distribution in this scenario and the hypothesis test cannot be performed as usual. However, the score test is still applicable in this scenario because it uses only the fit of the 2PL model. Accordingly, our approaches to power analysis can be reasonably applied only to the score test for this hypothesis type.

### Outlook

In the evaluation section, we focused on three alternative hypotheses that are relevant in practice; however, they represent only a fraction of the hypotheses available under the framework of linear hypotheses. Future studies may explore further alternatives, such as testing for DIF in more than one item simultaneously. The presented analytical framework as well as the R package implementation have therefore been designed for a straightforward extension to further application scenarios. In particular, we included templates for calculating the power for hypotheses using the 3PL model or multidimensional models (Reckase, 2009).

Another area of extension is the inclusion of further design parameters in the hypotheses. An example is the group variable in the DIF hypothesis. Kim et al. (1995) presented an approach to extend the Wald test to more than two groups and variable group sizes. Our approach can be easily extended to provide a power analysis for all four statistics in this scenario. Covariates can also influence the person parameter, e.g., when two groups have normally distributed ${\varvec{\theta }}$ parameters with different means. We can then estimate the means of the person parameter distribution separately for each group and calculate the statistics and their analytical power by including the parameters in the ${\varvec{\beta }}$ vector.

In our application of the linear hypothesis framework to investigate DIF, we used an implicit anchor to compare the parameter values of the first item. Specifically, we restricted the item parameters of the remaining items to be the same for both groups of test takers. To avoid misspecification, it is important to ensure that no DIF is present in these items, which might be difficult to establish in practice. One workaround is to estimate all items separately for both groups and apply an anchoring strategy afterward (Kopf et al., 2015). The interaction of such anchoring strategies and power may be the subject of further studies. The presented methods could be used to further investigate the general result that power increases with a higher number of items used in the anchor (Kopf et al., 2013).

One important step in an analysis of power is the selection of plausible and practically relevant deviations from the hypothesis or model of interest (Köhler & Hartig, 2017). There are usually multiple plausible alternative models against which the studied tests should have power. From the aspect of practical relevance, effect sizes must be considered. The researcher faces the task of selecting a suitable alternative model and thereby a relevant effect size. This must be done carefully, as the effect size should be large enough to represent a relevant violation, but also not so large that practically relevant violations might be overlooked when the sample size is chosen in accordance with it. A suitable discussion of this practical problem is given by Draxler (2010). Furthermore, there are general measures of difference between statistical models available (the Kullback–Leibler divergence, Kullback & Leibler, 1951). We can also consider specific item parameter sets and their distances from the respective null models as an effect size (Steinberg & Thissen, 2006). Yet, identical parameter differences can yield differences in the sample sizes required for detection since they are contingent on their absolute location. Thus, as noted by Draxler (2010), we might also consider the noncentrality parameters as an additional descriptor of effect size. Future studies are necessitated to further investigate the feasibility of the suggested effect sizes in IRT.

Although the gradient statistic is less established than the other three statistics (Draxler et al., 2020), we found that it exhibited the highest power in the scenarios of our simulation study. Hence, we call for further investigation of the pros and cons of the gradient statistic in IRT applications. This is especially relevant to questionnaires with higher numbers of items (e.g., 100), since calculating the gradient statistic generally involves the lowest computational effort of the four discussed statistics.

Finally, this work can be extended along the dimensions of the applied ML estimator and the considered statistics. Although we set a focus on MML estimation, the presented methods can also be considered for combination with other consistent ML estimators, e.g., pairwise maximum likelihood estimation (Katsikatsou et al., 2012). Another statistic to consider is the LR test by Andersen (1973). Although it is often used to evaluate overall model fit, an analytical power analysis is not yet available (Baker & Kim, 2004).

### Supplementary Information

Below is the link to the electronic supplementary material.

Supplementary file 1 (zip 115543 KB)

## Figures and Tables

**Fig. 12:**
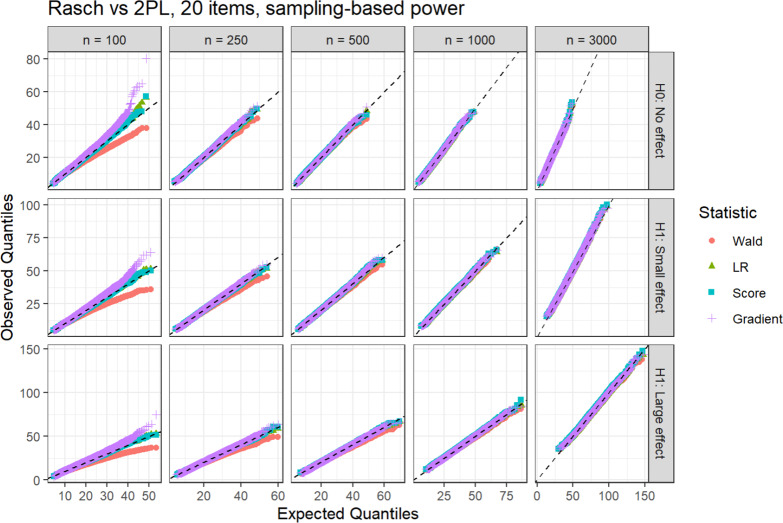
QQ-plots for the Rasch versus 2PL hypothesis with 20 items and the sampling-based power analysis method.

**Fig. 16:**
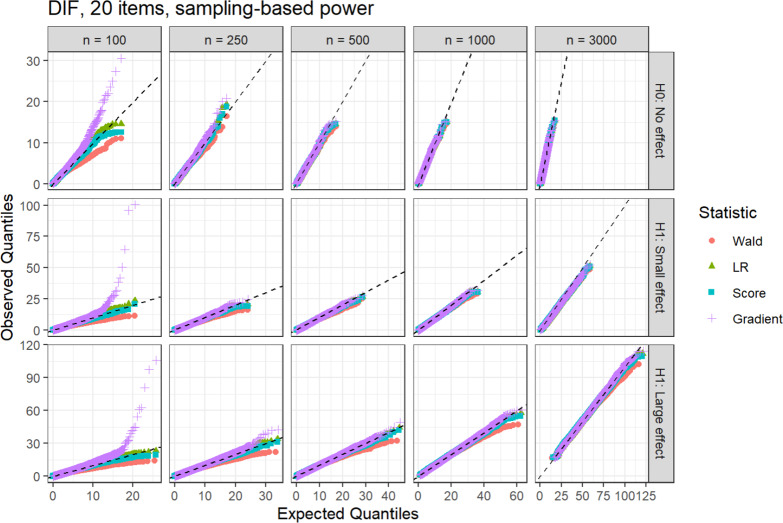
QQ-plots for the DIF hypothesis with 20 items and the sampling-based power analysis method.

**Fig. 17:**
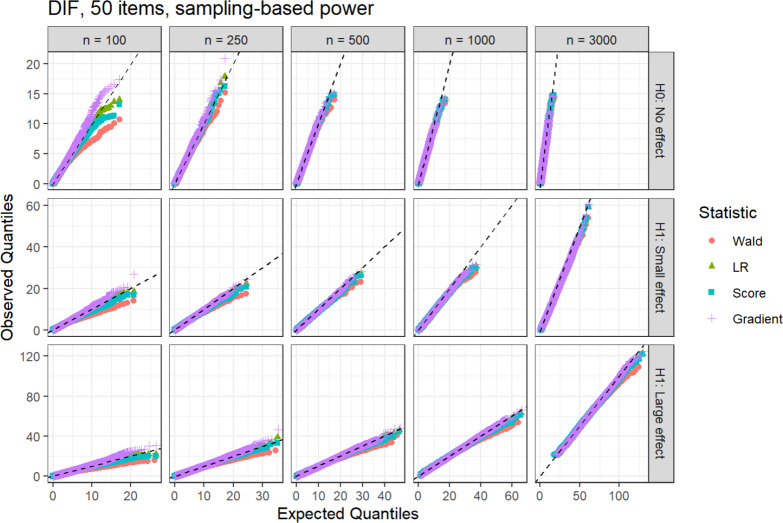
QQ-plots for the DIF hypothesis with 50 items and the sampling-based power analysis method.

**Fig. 22:**
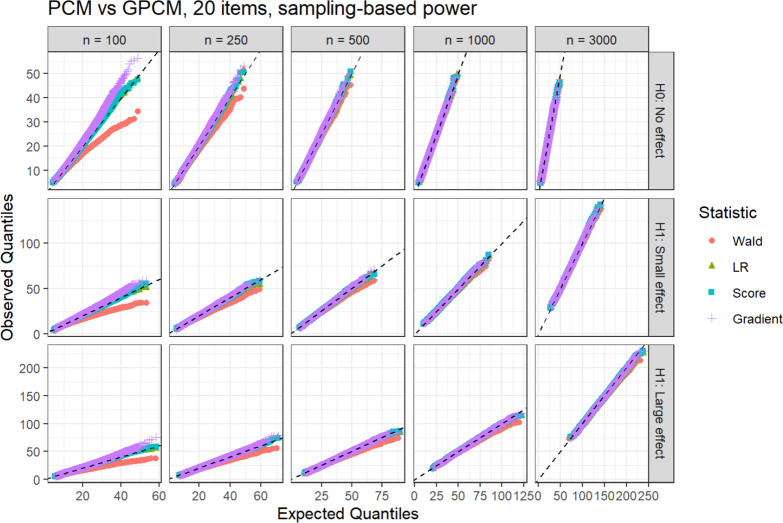
QQ-plots for the PCM versus GPCM hypothesis with 20 items and the sampling-based power analysis method.

**Fig. 23:**
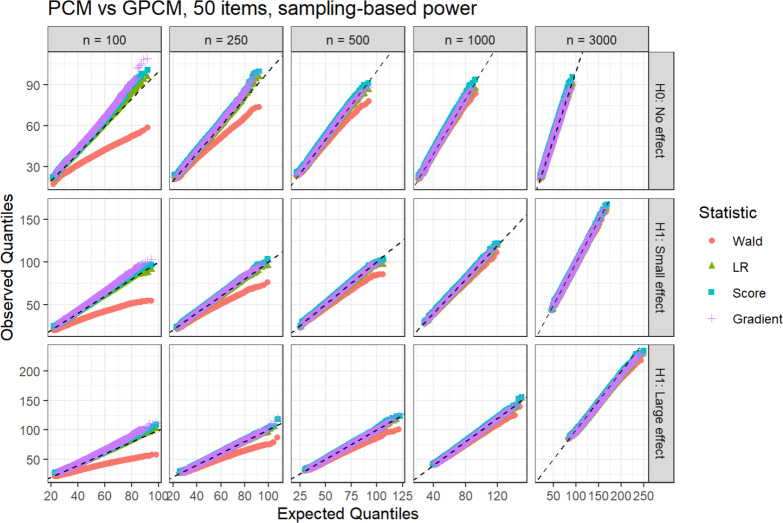
QQ-plots for the PCM versus GPCM hypothesis with 50 items and the sampling-based power analysis method.

**Fig. 24:**
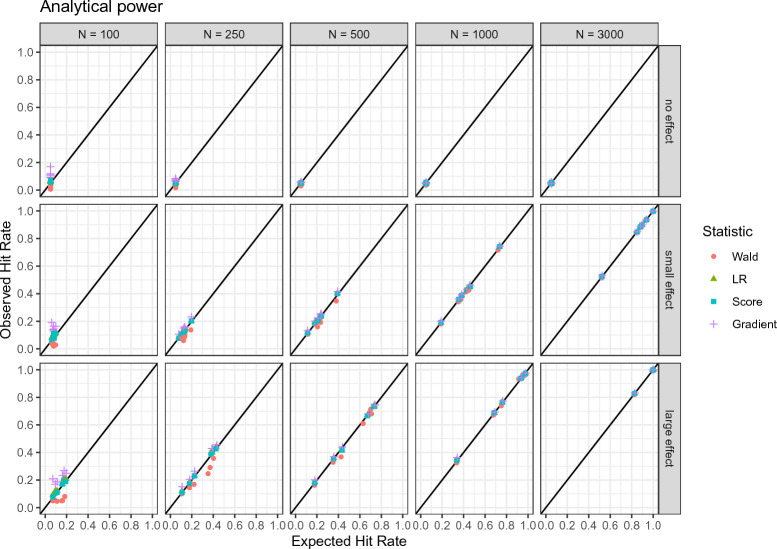
Observed and expected hit rates by effect size and sample size using the analytical power analysis approach.

**Fig. 25:**
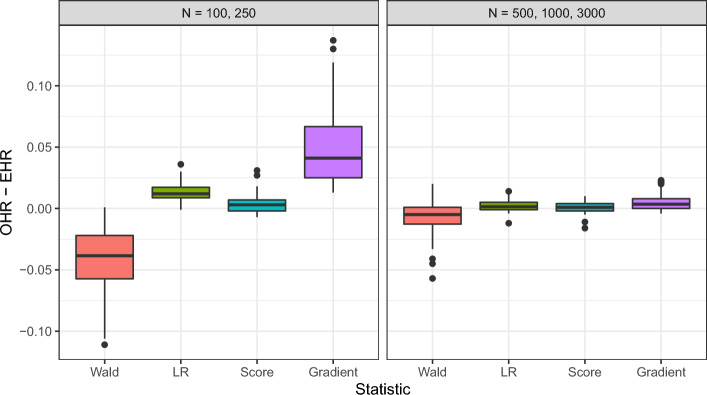
OHR-EHR: Observed minus expected hit rate. The expected hit rate was calculated using the analytical power analysis approach.

**Fig. 26:**
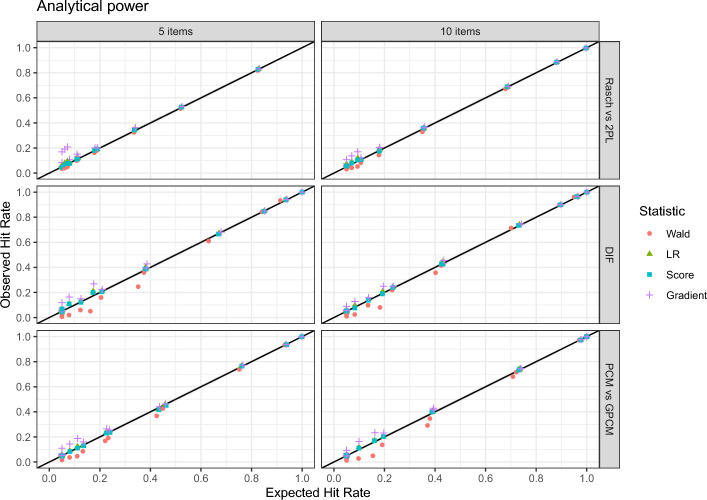
Observed and expected hit rates by hypothesis type and the number of items using the analytical power analysis approach.

## Appendix A

See Figs. 8, 9, 10, 11, 12, 13, 14, 15, 16, 17, 18, 19, 20, 21, 22 and 23.

## Appendix B

See Figs. 24, 25 and 26.

## Appendix C

See Tables 2, 3 and 4.

**Table 2:** Observed and expected hit rates for the Rasch versus 2PL hypothesis

				Wald			LR			Score			Gradient		
Items	Effect	*n*	Method	OHR	EHR	Env	OHR	EHR	Env	OHR	EHR	Env	OHR	EHR	Env
5	No	100	A	.036*	.050	[.042, .058]	.063*	.050	[.042, .058]	.046	.050	[.042, .058]	.169*	.050	[.042, .058]
5	No	250	A	.051	.050	[.042, .058]	.060*	.050	[.042, .058]	.048	.050	[.042, .058]	.085*	.050	[.042, .058]
5	No	500	A	.045	.050	[.042, .058]	.046	.050	[.042, .058]	.045	.050	[.042, .058]	.053	.050	[.042, .058]
5	No	1000	A	.052	.050	[.042, .058]	.050	.050	[.042, .058]	.049	.050	[.042, .058]	.053	.050	[.042, .058]
5	No	3000	A	.048	.050	[.042, .058]	.049	.050	[.042, .058]	.047	.050	[.042, .058]	.051	.050	[.042, .058]
5	Small	100	A	.040*	.061	[.053, .070]	.080*	.061	[.053, .070]	.064	.061	[.053, .070]	.191*	.061	[.053, .070]
5	Small	250	A	.074	.079	[.070, .089]	.084	.080	[.070, .090]	.077	.080	[.070, .089]	.107*	.080	[.070, .090]
5	Small	500	A	.106	.113	[.101, .124]	.120	.113	[.102, .125]	.116	.113	[.102, .125]	.133*	.114	[.102, .125]
5	Small	1000	A	.180	.188	[.174, .202]	.191	.189	[.175, .203]	.191	.189	[.174, .203]	.201	.190	[.175, .204]
5	Small	3000	A	.515	.520	[.502, .538]	.526	.523	[.505, .541]	.526	.522	[.503, .540]	.531	.525	[.507, .543]
5	Large	100	A	.050*	.072	[.062, .081]	.094*	.072	[.063, .081]	.077	.072	[.062, .081]	.209*	.072	[.063, .082]
5	Large	250	A	.100	.108	[.097, .120]	.121*	.109	[.098, .121]	.110	.109	[.097, .120]	.151*	.110	[.098, .121]
5	Large	500	A	.163*	.178	[.164, .192]	.186	.180	[.166, .194]	.179	.179	[.165, .193]	.202*	.181	[.167, .195]
5	Large	1000	A	.325	.335	[.318, .352]	.350	.338	[.321, .356]	.343	.337	[.319, .354]	.363*	.341	[.323, .358]
5	Large	3000	A	.819	.823	[.809, .837]	.830	.828	[.814, .842]	.827	.826	[.812, .839]	.832	.831	[.817, .844]
5	No	100	S	.036*	.050	[.042, .058]	.063*	.050	[.042, .058]	.046	.050	[.042, .058]	.169*	.050	[.042, .058]
5	No	250	S	.051	.050	[.042, .058]	.060*	.050	[.042, .058]	.048	.050	[.042, .058]	.085*	.050	[.042, .058]
5	No	500	S	.045	.050	[.042, .058]	.046	.050	[.042, .058]	.045	.050	[.042, .058]	.053	.050	[.042, .058]
5	No	1000	S	.052	.050	[.042, .058]	.050	.050	[.042, .058]	.049	.050	[.042, .058]	.053	.050	[.042, .058]
5	No	3000	S	.048	.050	[.042, .058]	.049	.050	[.042, .058]	.047	.050	[.042, .058]	.051	.050	[.042, .058]
5	Small	100	S	.040*	.061	[.053, .070]	.080*	.062	[.053, .070]	.064	.062	[.053, .070]	.191*	.062	[.053, .070]
5	Small	250	S	.074	.080	[.070, .090]	.084	.080	[.070, .090]	.077	.080	[.070, .090]	.107*	.080	[.071, .090]
5	Small	500	S	.106	.113	[.102, .125]	.120	.115	[.103, .126]	.116	.115	[.103, .126]	.133*	.115	[.103, .126]
5	Small	1000	S	.180	.189	[.175, .203]	.191	.192	[.178, .207]	.191	.192	[.178, .206]	.201	.193	[.178, .207]
5	Small	3000	S	.515	.522	[.504, .541]	.526	.532	[.514, .550]	.526	.531	[.513, .549]	.531	.533	[.515, .552]
5	Large	100	S	.050*	.072	[.062, .081]	.094*	.072	[.063, .081]	.077	.072	[.062, .081]	.209*	.072	[.063, .082]
5	Large	250	S	.100	.109	[.097, .120]	.121*	.109	[.098, .121]	.110	.108	[.097, .120]	.151*	.110	[.098, .121]
5	Large	500	S	.163*	.179	[.165, .193]	.186	.180	[.166, .194]	.179	.178	[.164, .192]	.202*	.181	[.167, .195]
5	Large	1000	S	.325	.336	[.319, .354]	.350	.339	[.321, .356]	.343	.335	[.318, .352]	.363*	.340	[.323, .358]
5	Large	3000	S	.819	.825	[.811, .839]	.830	.828	[.815, .842]	.827	.823	[.809, .837]	.832	.830	[.817, .844]
10	No	100	A	.032*	.050	[.042, .058]	.073*	.050	[.042, .058]	.057	.050	[.042, .058]	.108*	.050	[.042, .058]
10	No	250	A	.045	.050	[.042, .058]	.063*	.050	[.042, .058]	.054	.050	[.042, .058]	.075*	.050	[.042, .058]
10	No	500	A	.052	.050	[.042, .058]	.054	.050	[.042, .058]	.054	.050	[.042, .058]	.059*	.050	[.042, .058]
10	No	1000	A	.053	.050	[.042, .058]	.056	.050	[.042, .058]	.056	.050	[.042, .058]	.057	.050	[.042, .058]
10	No	3000	A	.051	.050	[.042, .058]	.052	.050	[.042, .058]	.053	.050	[.042, .058]	.051	.050	[.042, .058]
10	Small	100	A	.042*	.070	[.061, .080]	.090*	.071	[.061, .080]	.081*	.070	[.061, .080]	.137*	.071	[.061, .080]
10	Small	250	A	.083*	.106	[.095, .118]	.110	.107	[.096, .118]	.108	.107	[.096, .118]	.122*	.107	[.096, .118]
10	Small	500	A	.180	.179	[.165, .193]	.194	.180	[.166, .194]	.185	.180	[.166, .194]	.201*	.181	[.167, .195]
10	Small	1000	A	.341	.352	[.335, .370]	.357	.355	[.338, .373]	.358	.354	[.337, .372]	.362	.356	[.339, .374]
10	Small	3000	A	.880	.878	[.866, .890]	.885	.881	[.870, .893]	.885	.881	[.869, .892]	.888	.883	[.871, .894]
10	Large	100	A	.053*	.093	[.083, .104]	.120*	.094	[.084, .105]	.105*	.094	[.083, .105]	.170*	.094	[.084, .105]
10	Large	250	A	.144*	.178	[.164, .192]	.186	.181	[.167, .195]	.175	.180	[.166, .194]	.206*	.181	[.167, .195]
10	Large	500	A	.329*	.350	[.333, .367]	.360	.356	[.339, .374]	.354	.354	[.337, .372]	.368	.358	[.341, .375]
10	large	1000	A	.671	.679	[.662, .696]	.687	.688	[.671, .704]	.688	.685	[.668, .702]	.692	.690	[.674, .707]
10	Large	3000	A	.997	.997	[.995, .999]	.997	.998	[.996, .999]	.997	.998	[.996, .999]	.997	.998	[.996, .999]
10	No	100	S	.032*	.050	[.042, .058]	.073*	.050	[.042, .058]	.057	.050	[.042, .058]	.108*	.050	[.042, .058]
10	No	250	S	.045	.050	[.042, .058]	.063*	.050	[.042, .058]	.054	.050	[.042, .058]	.075*	.050	[.042, .058]
10	No	500	S	.052	.050	[.042, .058]	.054	.050	[.042, .058]	.054	.050	[.042, .058]	.059*	.050	[.042, .058]
10	No	1000	S	.053	.050	[.042, .058]	.056	.050	[.042, .058]	.056	.050	[.042, .058]	.057	.050	[.042, .058]
10	No	3000	S	.051	.050	[.042, .058]	.052	.050	[.042, .058]	.053	.050	[.042, .058]	.051	.050	[.042, .058]
10	Small	100	S	.042*	.071	[.062, .080]	.090*	.071	[.062, .081]	.081*	.071	[.062, .081]	.137*	.071	[.062, .081]
10	Small	250	S	.083*	.109	[.097, .120]	.110	.109	[.098, .120]	.108	.109	[.098, .120]	.122*	.109	[.098, .120]
10	Small	500	S	.180	.184	[.170, .198]	.194	.185	[.171, .199]	.185	.185	[.171, .199]	.201*	.185	[.171, .200]
10	Small	1000	S	.341*	.364	[.347, .382]	.357	.367	[.349, .384]	.358	.366	[.349, .384]	.362	.367	[.350, .385]
10	Small	3000	S	.880	.891	[.880, .902]	.885	.893	[.882, .904]	.885	.892	[.881, .904]	.888	.893	[.882, .905]
10	Large	100	S	.053*	.094	[.083, .105]	.120*	.095	[.084, .106]	.105	.095	[.084, .105]	.170*	.095	[.084, .106]
10	Large	250	S	.144*	.180	[.166, .194]	.186	.183	[.169, .197]	.175	.182	[.168, .196]	.206*	.183	[.169, .197]
10	Large	500	S	.329*	.356	[.338, .373]	.360	.361	[.344, .379]	.354	.359	[.342, .377]	.368	.363	[.345, .380]
10	Large	1000	S	.671	.687	[.670, .704]	.687	.695	[.678, .712]	.688	.692	[.675, .709]	.692	.697	[.680, .714]
10	Large	3000	S	.997	.998	[.996, .999]	.997	.998	[.996, 1.000]	.997	.998	[.996, 1.000]	.997	.998	[.996, 1.000]
20	No	100	S	.009*	.050	[.042, .058]	.068*	.050	[.042, .058]	.059*	.050	[.042, .058]	.096*	.050	[.042, .058]
20	No	250	S	.031*	.050	[.042, .058]	.059*	.050	[.042, .058]	.060*	.050	[.042, .058]	.068*	.050	[.042, .058]
20	No	500	S	.038*	.050	[.042, .058]	.054	.050	[.042, .058]	.051	.050	[.042, .058]	.057	.050	[.042, .058]
20	No	1000	S	.047	.050	[.042, .058]	.057	.050	[.042, .058]	.058	.050	[.042, .058]	.058	.050	[.042, .058]
20	No	3000	S	.046	.050	[.042, .058]	.049	.050	[.042, .058]	.050	.050	[.042, .058]	.050	.050	[.042, .058]
20	Small	100	S	.010*	.068	[.059, .078]	.089*	.068	[.059, .078]	.078*	.068	[.059, .078]	.126*	.068	[.059, .078]
20	Small	250	S	.058*	.102	[.091, .113]	.109	.102	[.091, .113]	.108	.102	[.091, .113]	.125*	.102	[.091, .113]
20	Small	500	S	.146*	.171	[.158, .185]	.183	.172	[.158, .186]	.182	.171	[.158, .185]	.191*	.173	[.159, .186]
20	Small	1000	S	.338	.348	[.330, .365]	.367	.350	[.333, .367]	.369*	.348	[.330, .365]	.371*	.351	[.334, .368]
20	Small	3000	S	.906	.900	[.889, .911]	.914*	.903	[.892, .913]	.912*	.900	[.889, .911]	.915*	.904	[.893, .914]
20	Large	100	S	.021*	.096	[.085, .106]	.122*	.097	[.086, .107]	.106	.096	[.085, .107]	.159*	.097	[.086, .108]
20	Large	250	S	.135*	.191	[.177, .206]	.217*	.195	[.181, .209]	.213*	.193	[.179, .207]	.230*	.196	[.181, .210]
20	Large	500	S	.365*	.396	[.378, .414]	.425*	.405	[.387, .422]	.422*	.400	[.382, .418]	.435*	.407	[.389, .425]
20	Large	1000	S	.761	.766	[.751, .782]	.786	.777	[.762, .792]	.779	.771	[.756, .786]	.787	.780	[.764, .795]
20	Large	3000	S	1.000	1.000	[.999, 1.000]	1.000	1.000	[.999, 1.000]	1.000	1.000	[.999, 1.000]	1.000	1.000	[.999, 1.000]
50	No	100	S	.000*	.050	[.042, .058]	.068*	.050	[.042, .058]	.060*	.050	[.042, .058]	.109*	.050	[.042, .058]
50	No	250	S	.006*	.050	[.042, .058]	.060*	.050	[.042, .058]	.056	.050	[.042, .058]	.072*	.050	[.042, .058]
50	No	500	S	.019*	.050	[.042, .058]	.055	.050	[.042, .058]	.055	.050	[.042, .058]	.060*	.050	[.042, .058]
50	No	1000	S	.031*	.050	[.042, .058]	.052	.050	[.042, .058]	.053	.050	[.042, .058]	.055	.050	[.042, .058]
50	No	3000	S	.042*	.050	[.042, .058]	.049	.050	[.042, .058]	.049	.050	[.042, .058]	.049	.050	[.042, .058]
50	Small	100	S	.000*	.066	[.057, .075]	.096*	.066	[.057, .075]	.083*	.066	[.057, .075]	.142*	.066	[.057, .075]
50	Small	250	S	.011*	.094	[.083, .105]	.101	.095	[.084, .105]	.100	.094	[.084, .105]	.118*	.095	[.084, .105]
50	Small	500	S	.067*	.155	[.142, .168]	.153	.157	[.144, .170]	.151	.156	[.143, .169]	.165	.157	[.144, .170]
50	Small	1000	S	.244*	.319	[.303, .336]	.314	.324	[.307, .341]	.314	.323	[.306, .340]	.322	.325	[.308, .342]
50	Small	3000	S	.877*	.901	[.890, .912]	.890*	.906	[.896, .917]	.890*	.904	[.894, .915]	.891*	.907	[.896, .917]
50	Large	100	S	.000*	.118	[.106, .130]	.169*	.121	[.109, .133]	.151*	.120	[.108, .132]	.227*	.122	[.110, .133]
50	Large	250	S	.083*	.280	[.264, .297]	.333*	.292	[.276, .309]	.320*	.288	[.272, .305]	.363*	.294	[.277, .310]
50	Large	500	S	.492*	.611	[.593, .629]	.666*	.634	[.616, .651]	.663*	.626	[.609, .644]	.682*	.636	[.619, .654]
50	Large	1000	S	.959	.956	[.949, .964]	.977*	.965	[.958, .971]	.975*	.962	[.955, .969]	.979*	.965	[.959, .972]
50	Large	3000	S	1.000	1.000	[1.000, 1.000]	1.000	1.000	[1.000, 1.000]	1.000	1.000	[1.000, 1.000]	1.000	1.000	[1.000, 1.000]

*A* analytical method, *S* sampling-based method, *n* sample size, *OHR* observed hit rate, *EHR* expected hit rate, *Env* 99% confidence envelope of the expected hit rate. OHR’s that lie outside of the envelope are marked with a star.

**Table 3:** Observed and expected hit rates for the DIF hypothesis

				Wald			LR			Score			Gradient		
Items	Effect	*n*	Method	OHR	EHR	Env	OHR	EHR	Env	OHR	EHR	Env	OHR	EHR	Env
5	No	100	A	.006*	.050	[.042, .058]	.067*	.050	[.042, .058]	.068*	.050	[.042, .058]	.119*	.050	[.042, .058]
5	No	250	A	.017*	.050	[.042, .058]	.060*	.050	[.042, .058]	.052	.050	[.042, .058]	.076*	.050	[.042, .058]
5	No	500	A	.030*	.050	[.042, .058]	.056	.050	[.042, .058]	.052	.050	[.042, .058]	.059*	.050	[.042, .058]
5	No	1000	A	.035*	.050	[.042, .058]	.047	.050	[.042, .058]	.045	.050	[.042, .058]	.049	.050	[.042, .058]
5	No	3000	A	.052	.050	[.042, .058]	.057	.050	[.042, .058]	.056	.050	[.042, .058]	.058*	.050	[.042, .058]
5	Small	100	A	.020*	.078	[.068, .088]	.108*	.078	[.069, .088]	.109*	.078	[.069, .088]	.164*	.079	[.069, .088]
5	Small	250	A	.060*	.123	[.111, .135]	.133	.125	[.113, .137]	.122	.125	[.113, .137]	.150*	.125	[.113, .137]
5	Small	500	A	.160*	.205	[.190, .219]	.214	.209	[.194, .223]	.206	.208	[.194, .223]	.224*	.209	[.195, .224]
5	Small	1000	A	.359	.375	[.357, .392]	.390	.382	[.365, .400]	.387	.382	[.364, .400]	.394	.384	[.366, .402]
5	Small	3000	A	.844	.841	[.828, .855]	.849	.850	[.837, .863]	.848	.850	[.837, .863]	.850	.852	[.839, .865]
5	Large	100	A	.051*	.162	[.149, .176]	.210*	.174	[.160, .187]	.200*	.173	[.159, .187]	.268*	.176	[.162, .190]
5	Large	250	A	.246*	.352	[.334, .369]	.401*	.381	[.363, .398]	.386	.379	[.361, .396]	.428*	.387	[.369, .404]
5	Large	500	A	.610*	.630	[.613, .648]	.673	.672	[.654, .689]	.666	.669	[.652, .686]	.682	.680	[.663, .697]
5	Large	1000	A	.934*	.914	[.903, .924]	.941	.936	[.927, .945]	.939	.935	[.926, .944]	.942	.940	[.932, .949]
5	Large	3000	A	1.000	1.000	[1.000, 1.000]	1.000	1.000	[1.000, 1.000]	1.000	1.000	[1.000, 1.000]	1.000	1.000	[1.000, 1.000]
5	No	100	S	.006*	.050	[.042, .058]	.067*	.050	[.042, .058]	.068*	.050	[.042, .058]	.119*	.050	[.042, .058]
5	No	250	S	.017*	.050	[.042, .058]	.060*	.050	[.042, .058]	.052	.050	[.042, .058]	.076*	.050	[.042, .058]
5	No	500	S	.030*	.050	[.042, .058]	.056	.050	[.042, .058]	.052	.050	[.042, .058]	.059*	.050	[.042, .058]
5	No	1000	S	.035*	.050	[.042, .058]	.047	.050	[.042, .058]	.045	.050	[.042, .058]	.049	.050	[.042, .058]
5	No	3000	S	.052	.050	[.042, .058]	.057	.050	[.042, .058]	.056	.050	[.042, .058]	.058*	.050	[.042, .058]
5	Small	100	S	.020*	.077	[.067, .086]	.108*	.077	[.068, .087]	.109*	.077	[.068, .087]	.164*	.077	[.068, .087]
5	Small	250	S	.060*	.120	[.108, .132]	.133	.122	[.110, .134]	.122	.122	[.110, .134]	.150*	.122	[.110, .134]
5	Small	500	S	.160*	.198	[.184, .213]	.214	.202	[.187, .216]	.206	.202	[.187, .216]	.224*	.202	[.188, .217]
5	Small	1000	S	.359	.362	[.344, .379]	.390*	.369	[.351, .387]	.387*	.369	[.351, .386]	.394*	.370	[.353, .388]
5	Small	3000	S	.844*	.826	[.812, .840]	.849*	.835	[.821, .848]	.848	.835	[.821, .848]	.850	.836	[.823, .850]
5	Large	100	S	.051*	.159	[.146, .173]	.210*	.170	[.157, .184]	.200*	.170	[.156, .184]	.268*	.173	[.159, .186]
5	Large	250	S	.246*	.344	[.327, .361]	.401*	.373	[.355, .390]	.386	.371	[.354, .389]	.428*	.378	[.360, .396]
5	Large	500	S	.610	.619	[.601, .637]	.673	.660	[.643, .678]	.666	.659	[.642, .676]	.682	.668	[.651, .685]
5	Large	1000	S	.934*	.907	[.896, .917]	.941*	.931	[.921, .940]	.939	.930	[.920, .939]	.942	.935	[.926, .944]
5	Large	3000	S	1.000	1.000	[1.000, 1.000]	1.000	1.000	[1.000, 1.000]	1.000	1.000	[1.000, 1.000]	1.000	1.000	[1.000, 1.000]
10	No	100	A	.010*	.050	[.042, .058]	.062*	.050	[.042, .058]	.050	.050	[.042, .058]	.089*	.050	[.042, .058]
10	No	250	A	.028*	.050	[.042, .058]	.054	.050	[.042, .058]	.049	.050	[.042, .058]	.063*	.050	[.042, .058]
10	No	500	A	.039*	.050	[.042, .058]	.051	.050	[.042, .058]	.048	.050	[.042, .058]	.055	.050	[.042, .058]
10	No	1000	A	.049	.050	[.042, .058]	.055	.050	[.042, .058]	.054	.050	[.042, .058]	.056	.050	[.042, .058]
10	No	3000	A	.045	.050	[.042, .058]	.046	.050	[.042, .058]	.046	.050	[.042, .058]	.046	.050	[.042, .058]
10	Small	100	A	.025*	.082	[.072, .092]	.099*	.083	[.073, .093]	.076	.083	[.073, .093]	.128*	.083	[.073, .093]
10	Small	250	A	.098*	.135	[.122, .147]	.148	.137	[.124, .149]	.138	.136	[.124, .149]	.159*	.137	[.124, .149]
10	Small	500	A	.220	.230	[.215, .245]	.245	.234	[.218, .249]	.238	.233	[.218, .249]	.250	.235	[.219, .250]
10	Small	1000	A	.415	.424	[.406, .442]	.430	.431	[.413, .449]	.426	.430	[.412, .448]	.433	.433	[.415, .451]
10	Small	3000	A	.898	.891	[.879, .902]	.900	.897	[.886, .908]	.899	.896	[.885, .907]	.900	.898	[.887, .909]
10	Large	100	A	.081*	.182	[.168, .196]	.209*	.193	[.179, .207]	.189	.192	[.178, .206]	.250*	.196	[.181, .210]
10	Large	250	A	.357*	.402	[.384, .420]	.443	.428	[.410, .446]	.428	.426	[.408, .444]	.456*	.435	[.417, .453]
10	Large	500	A	.714	.701	[.684, .717]	.741	.734	[.718, .750]	.735	.730	[.714, .746]	.746	.741	[.725, .757]
10	Large	1000	A	.961*	.950	[.942, .958]	.964	.963	[.956, .970]	.964	.961	[.954, .968]	.964	.965	[.959, .972]
10	Large	3000	A	1.000*	1.000	[1.000, 1.000]	1.000*	1.000	[1.000, 1.000]	1.000*	1.000	[1.000, 1.000]	1.000*	1.000	[1.000, 1.000]
10	No	100	S	.010*	.050	[.042, .058]	.062*	.050	[.042, .058]	.050	.050	[.042, .058]	.089*	.050	[.042, .058]
10	No	250	S	.028*	.050	[.042, .058]	.054	.050	[.042, .058]	.049	.050	[.042, .058]	.063*	.050	[.042, .058]
10	No	500	S	.039*	.050	[.042, .058]	.051	.050	[.042, .058]	.048	.050	[.042, .058]	.055	.050	[.042, .058]
10	No	1000	S	.049	.050	[.042, .058]	.055	.050	[.042, .058]	.054	.050	[.042, .058]	.056	.050	[.042, .058]
10	No	3000	S	.045	.050	[.042, .058]	.046	.050	[.042, .058]	.046	.050	[.042, .058]	.046	.050	[.042, .058]
10	Small	100	S	.025*	.084	[.074, .094]	.099*	.084	[.074, .094]	.076	.084	[.074, .094]	.128*	.085	[.074, .095]
10	Small	250	S	.098*	.139	[.126, .152]	.148	.141	[.128, .154]	.138	.141	[.128, .153]	.159*	.141	[.129, .154]
10	Small	500	S	.220*	.239	[.223, .254]	.245	.243	[.227, .259]	.238	.243	[.227, .258]	.250	.244	[.228, .260]
10	Small	1000	S	.415*	.441	[.423, .459]	.430*	.448	[.430, .467]	.426*	.448	[.430, .466]	.433	.450	[.432, .468]
10	Small	3000	S	.898	.904	[.894, .915]	.900*	.910	[.900, .920]	.899	.910	[.899, .920]	.900*	.911	[.901, .922]
10	Large	100	S	.081*	.183	[.169, .197]	.209*	.194	[.179, .208]	.189	.193	[.178, .207]	.250*	.196	[.182, .211]
10	Large	250	S	.357*	.404	[.386, .422]	.443	.430	[.412, .448]	.428	.427	[.409, .445]	.456*	.437	[.419, .455]
10	Large	500	S	.714	.703	[.687, .720]	.741	.736	[.720, .752]	.735	.732	[.716, .748]	.746	.743	[.728, .759]
10	Large	1000	S	.961*	.951	[.943, .959]	.964	.963	[.957, .970]	.964	.962	[.955, .969]	.964	.966	[.959, .973]
10	Large	3000	S	1.000*	1.000	[1.000, 1.000]	1.000*	1.000	[1.000, 1.000]	1.000*	1.000	[1.000, 1.000]	1.000*	1.000	[1.000, 1.000]
20	No	100	S	.016*	.050	[.042, .058]	.059*	.050	[.042, .058]	.048	.050	[.042, .058]	.078*	.050	[.042, .058]
20	No	250	S	.033*	.050	[.042, .058]	.048	.050	[.042, .058]	.044	.050	[.042, .058]	.054	.050	[.042, .058]
20	No	500	S	.038*	.050	[.042, .058]	.045	.050	[.042, .058]	.044	.050	[.042, .058]	.046	.050	[.042, .058]
20	No	1000	S	.048	.050	[.042, .058]	.050	.050	[.042, .058]	.049	.050	[.042, .058]	.051	.050	[.042, .058]
20	No	3000	S	.049	.050	[.042, .058]	.051	.050	[.042, .058]	.051	.050	[.042, .058]	.052	.050	[.042, .058]
20	Small	100	S	.036*	.092	[.082, .103]	.098	.093	[.082, .104]	.081*	.093	[.083, .104]	.113*	.093	[.083, .104]
20	Small	250	S	.113*	.162	[.149, .176]	.148*	.165	[.151, .178]	.140*	.165	[.152, .179]	.155	.165	[.152, .179]
20	Small	500	S	.249*	.289	[.272, .305]	.269*	.293	[.276, .310]	.263*	.294	[.277, .311]	.274*	.294	[.277, .311]
20	Small	1000	S	.469*	.530	[.512, .548]	.477*	.537	[.519, .555]	.478*	.539	[.521, .557]	.481*	.539	[.521, .557]
20	Small	3000	S	.927*	.956	[.949, .963]	.928*	.959	[.952, .966]	.928*	.960	[.953, .967]	.928*	.960	[.953, .967]
20	Large	100	S	.116*	.199	[.185, .214]	.236*	.209	[.194, .223]	.213	.207	[.193, .222]	.261*	.211	[.196, .226]
20	Large	250	S	.426	.443	[.425, .461]	.476	.465	[.447, .483]	.464	.462	[.444, .480]	.488	.471	[.453, .489]
20	Large	500	S	.771*	.751	[.735, .766]	.786	.776	[.761, .791]	.782	.773	[.757, .788]	.790	.782	[.767, .797]
20	Large	1000	S	.976*	.968	[.962, .975]	.977	.976	[.970, .981]	.977	.975	[.969, .981]	.977	.978	[.972, .983]
20	Large	3000	S	1.000	1.000	[1.000, 1.000]	1.000	1.000	[1.000, 1.000]	1.000	1.000	[1.000, 1.000]	1.000	1.000	[1.000, 1.000]
50	No	100	S	.024*	.050	[.042, .058]	.058*	.050	[.042, .058]	.049	.050	[.042, .058]	.068*	.050	[.042, .058]
50	No	250	S	.043	.050	[.042, .058]	.055	.050	[.042, .058]	.051	.050	[.042, .058]	.057	.050	[.042, .058]
50	No	500	S	.047	.050	[.042, .058]	.052	.050	[.042, .058]	.050	.050	[.042, .058]	.053	.050	[.042, .058]
50	No	1000	S	.041*	.050	[.042, .058]	.043	.050	[.042, .058]	.042*	.050	[.042, .058]	.043	.050	[.042, .058]
50	No	3000	S	.047	.050	[.042, .058]	.048	.050	[.042, .058]	.048	.050	[.042, .058]	.048	.050	[.042, .058]
50	Small	100	S	.051*	.095	[.085, .106]	.100	.097	[.086, .107]	.085*	.096	[.085, .107]	.118*	.097	[.086, .107]
50	Small	250	S	.138*	.171	[.158, .185]	.158*	.174	[.161, .188]	.153*	.174	[.160, .187]	.166	.175	[.161, .189]
50	Small	500	S	.266*	.307	[.291, .324]	.281*	.313	[.296, .330]	.277*	.312	[.295, .329]	.286*	.314	[.298, .331]
50	Small	1000	S	.502*	.561	[.543, .579]	.510*	.571	[.553, .589]	.508*	.568	[.550, .586]	.512*	.573	[.555, .591]
50	Small	3000	S	.950*	.968	[.961, .974]	.950*	.971	[.965, .977]	.949*	.970	[.964, .976]	.950*	.971	[.965, .977]
50	Large	100	S	.149*	.217	[.202, .232]	.234	.227	[.212, .242]	.209*	.226	[.210, .241]	.254*	.230	[.214, .245]
50	Large	250	S	.481	.484	[.466, .502]	.510	.507	[.488, .525]	.501	.504	[.486, .522]	.519	.513	[.495, .531]
50	Large	500	S	.807	.796	[.781, .811]	.819	.818	[.804, .832]	.816	.816	[.801, .830]	.823	.824	[.810, .838]
50	Large	1000	S	.986	.981	[.976, .986]	.987	.986	[.981, .990]	.987	.985	[.981, .990]	.987	.987	[.983, .991]
50	Large	3000	S	1.000	1.000	[1.000, 1.000]	1.000	1.000	[1.000, 1.000]	1.000	1.000	[1.000, 1.000]	1.000	1.000	[1.000, 1.000]

*A* analytical method, *S* sampling-based method, *n* sample size, *OHR* observed hit rate, *EHR* expected hit rate, *Env* 99% confidence envelope of the expected hit rate. OHR’s that lie outside of the envelope are marked with a star.

**Table 4:** Observed and expected hit rates for the PCM versus GPCM hypothesis

				Wald			LR			Score			Gradient		
Items	Effect	*n*	Method	OHR	EHR	Env	OHR	EHR	Env	OHR	EHR	Env	OHR	EHR	Env
5	No	100	A	.017*	.050	[.042, .058]	.059*	.050	[.042, .058]	.054	.050	[.042, .058]	.109*	.050	[.042, .058]
5	No	250	A	.033*	.050	[.042, .058]	.049	.050	[.042, .058]	.046	.050	[.042, .058]	.065*	.050	[.042, .058]
5	No	500	A	.038*	.050	[.042, .058]	.051	.050	[.042, .058]	.052	.050	[.042, .058]	.057	.050	[.042, .058]
5	No	1000	A	.041*	.050	[.042, .058]	.048	.050	[.042, .058]	.047	.050	[.042, .058]	.052	.050	[.042, .058]
5	No	3000	A	.049	.050	[.042, .058]	.052	.050	[.042, .058]	.052	.050	[.042, .058]	.052	.050	[.042, .058]
5	Small	100	A	.037*	.080	[.070, .090]	.090	.081	[.071, .091]	.084	.081	[.071, .091]	.144*	.081	[.071, .091]
5	Small	250	A	.086*	.133	[.120, .145]	.135	.134	[.122, .147]	.130	.136	[.123, .148]	.160*	.135	[.123, .147]
5	Small	500	A	.192*	.233	[.218, .249]	.239	.237	[.222, .253]	.236	.240	[.225, .256]	.255*	.239	[.223, .254]
5	Small	1000	A	.426*	.449	[.431, .467]	.455	.457	[.439, .475]	.451	.462	[.444, .480]	.462	.460	[.441, .478]
5	Small	3000	A	.933	.931	[.922, .940]	.937	.936	[.927, .945]	.936	.939	[.930, .948]	.937	.937	[.929, .946]
5	Large	100	A	.046*	.110	[.099, .122]	.123*	.111	[.100, .123]	.110	.112	[.100, .123]	.187*	.112	[.101, .124]
5	Large	250	A	.169*	.221	[.206, .236]	.235	.225	[.209, .240]	.230	.225	[.210, .241]	.264*	.227	[.212, .242]
5	Large	500	A	.368*	.425	[.407, .443]	.420	.432	[.414, .450]	.417	.433	[.415, .451]	.440	.436	[.418, .454]
5	Large	1000	A	.739	.752	[.736, .767]	.765	.760	[.744, .775]	.764	.762	[.746, .777]	.773	.765	[.750, .781]
5	Large	3000	A	.999	.998	[.997, 1.000]	.999	.999	[.997, 1.000]	.999	.999	[.997, 1.000]	.999	.999	[.998, 1.000]
5	No	100	S	.017*	.050	[.042, .058]	.059*	.050	[.042, .058]	.054	.050	[.042, .058]	.109*	.050	[.042, .058]
5	No	250	S	.033*	.050	[.042, .058]	.049	.050	[.042, .058]	.046	.050	[.042, .058]	.065*	.050	[.042, .058]
5	No	500	S	.038*	.050	[.042, .058]	.051	.050	[.042, .058]	.052	.050	[.042, .058]	.057	.050	[.042, .058]
5	No	1000	S	.041*	.050	[.042, .058]	.048	.050	[.042, .058]	.047	.050	[.042, .058]	.052	.050	[.042, .058]
5	No	3000	S	.049	.050	[.042, .058]	.052	.050	[.042, .058]	.052	.050	[.042, .058]	.052	.050	[.042, .058]
5	Small	100	S	.037*	.080	[.070, .090]	.090	.081	[.071, .091]	.084	.081	[.071, .090]	.144*	.081	[.071, .091]
5	Small	250	S	.086*	.133	[.120, .145]	.135	.134	[.122, .147]	.130	.134	[.121, .146]	.160*	.135	[.123, .147]
5	Small	500	S	.192*	.234	[.218, .249]	.239	.237	[.221, .252]	.236	.236	[.220, .251]	.255*	.239	[.223, .254]
5	Small	1000	S	.426*	.450	[.432, .468]	.455	.456	[.438, .474]	.451	.454	[.436, .472]	.462	.459	[.441, .477]
5	Small	3000	S	.933	.932	[.922, .941]	.937	.935	[.926, .944]	.936	.934	[.925, .943]	.937	.937	[.928, .946]
5	Large	100	S	.046*	.107	[.096, .119]	.123*	.109	[.098, .121]	.110	.109	[.098, .121]	.187*	.110	[.099, .122]
5	Large	250	S	.169*	.213	[.198, .228]	.235*	.219	[.204, .234]	.230	.218	[.203, .233]	.264*	.221	[.206, .236]
5	Large	500	S	.368*	.408	[.390, .426]	.420	.420	[.402, .438]	.417	.419	[.401, .437]	.440	.425	[.407, .443]
5	Large	1000	S	.739	.731	[.715, .747]	.765*	.746	[.730, .762]	.764*	.745	[.729, .761]	.773*	.752	[.736, .768]
5	Large	3000	S	.999	.998	[.996, .999]	.999	.998	[.997, 1.000]	.999	.998	[.997, 1.000]	.999	.998	[.997, 1.000]
10	No	100	A	.013*	.050	[.042, .058]	.061*	.050	[.042, .058]	.055	.050	[.042, .058]	.093*	.050	[.042, .058]
10	No	250	A	.028*	.050	[.042, .058]	.054	.050	[.042, .058]	.054	.050	[.042, .058]	.066*	.050	[.042, .058]
10	No	500	A	.035*	.050	[.042, .058]	.050	.050	[.042, .058]	.052	.050	[.042, .058]	.056	.050	[.042, .058]
10	No	1000	A	.043	.050	[.042, .058]	.047	.050	[.042, .058]	.048	.050	[.042, .058]	.048	.050	[.042, .058]
10	No	3000	A	.045	.050	[.042, .058]	.046	.050	[.042, .058]	.047	.050	[.042, .058]	.047	.050	[.042, .058]
10	Small	100	A	.028*	.097	[.087, .108]	.117*	.099	[.088, .110]	.111*	.099	[.088, .110]	.163*	.099	[.088, .110]
10	Small	250	A	.137*	.191	[.177, .205]	.203	.196	[.181, .210]	.202	.196	[.181, .210]	.231*	.197	[.182, .211]
10	Small	500	A	.347*	.380	[.362, .397]	.402	.391	[.373, .408]	.400	.390	[.372, .408]	.416*	.393	[.375, .411]
10	Small	1000	A	.716	.721	[.705, .737]	.742	.735	[.719, .752]	.741	.735	[.719, .751]	.744	.739	[.723, .755]
10	Small	3000	A	.999	.999	[.997, 1.000]	.999	.999	[.998, 1.000]	.999	.999	[.998, 1.000]	.999	.999	[.998, 1.000]
10	Large	100	A	.049*	.154	[.141, .167]	.171	.161	[.147, .174]	.170	.160	[.147, .173]	.234*	.162	[.149, .176]
10	Large	250	A	.291*	.370	[.353, .388]	.401	.389	[.372, .407]	.400	.388	[.370, .405]	.429*	.394	[.376, .412]
10	Large	500	A	.681*	.708	[.691, .724]	.733	.734	[.718, .750]	.733	.732	[.716, .748]	.745	.740	[.724, .756]
10	Large	1000	A	.971	.970	[.964, .976]	.975	.977	[.972, .982]	.973	.976	[.971, .982]	.976	.979	[.973, .984]
10	Large	3000	A	1.000	1.000	[1.000, 1.000]	1.000	1.000	[1.000, 1.000]	1.000	1.000	[1.000, 1.000]	1.000	1.000	[1.000, 1.000]
10	No	100	S	.013*	.050	[.042, .058]	.061*	.050	[.042, .058]	.055	.050	[.042, .058]	.093*	.050	[.042, .058]
10	No	250	S	.028*	.050	[.042, .058]	.054	.050	[.042, .058]	.054	.050	[.042, .058]	.066*	.050	[.042, .058]
10	No	500	S	.035*	.050	[.042, .058]	.050	.050	[.042, .058]	.052	.050	[.042, .058]	.056	.050	[.042, .058]
10	No	1000	S	.043	.050	[.042, .058]	.047	.050	[.042, .058]	.048	.050	[.042, .058]	.048	.050	[.042, .058]
10	No	3000	S	.045	.050	[.042, .058]	.046	.050	[.042, .058]	.047	.050	[.042, .058]	.047	.050	[.042, .058]
10	Small	100	S	.028*	.097	[.086, .108]	.117*	.098	[.087, .109]	.111*	.098	[.087, .109]	.163*	.099	[.088, .109]
10	Small	250	S	.137*	.190	[.176, .204]	.203	.194	[.179, .208]	.202	.194	[.179, .208]	.231*	.194	[.180, .209]
10	Small	500	S	.347*	.378	[.360, .395]	.402	.385	[.368, .403]	.400	.385	[.368, .403]	.416*	.387	[.370, .405]
10	Small	1000	S	.716	.718	[.702, .735]	.742	.729	[.712, .745]	.741	.729	[.712, .745]	.744	.731	[.715, .748]
10	Small	3000	S	.999	.999	[.997, 1.000]	.999	.999	[.998, 1.000]	.999	.999	[.998, 1.000]	.999	.999	[.998, 1.000]
10	Large	100	S	.049*	.154	[.141, .167]	.171	.160	[.147, .174]	.170	.160	[.146, .173]	.234*	.162	[.149, .175]
10	Large	250	S	.291*	.371	[.353, .388]	.401	.388	[.371, .406]	.400	.387	[.369, .405]	.429*	.394	[.376, .412]
10	Large	500	S	.681*	.708	[.692, .725]	.733	.732	[.716, .749]	.733	.731	[.715, .747]	.745	.740	[.724, .756]
10	Large	1000	S	.971	.970	[.964, .976]	.975	.977	[.971, .982]	.973	.976	[.971, .982]	.976	.978	[.973, .984]
10	Large	3000	S	1.000	1.000	[1.000, 1.000]	1.000	1.000	[1.000, 1.000]	1.000	1.000	[1.000, 1.000]	1.000	1.000	[1.000, 1.000]
20	No	100	S	.001*	.050	[.042, .058]	.063*	.050	[.042, .058]	.061*	.050	[.042, .058]	.105*	.050	[.042, .058]
20	No	250	S	.022*	.050	[.042, .058]	.060*	.050	[.042, .058]	.058*	.050	[.042, .058]	.075*	.050	[.042, .058]
20	No	500	S	.031*	.050	[.042, .058]	.054	.050	[.042, .058]	.055	.050	[.042, .058]	.061*	.050	[.042, .058]
20	No	1000	S	.043	.050	[.042, .058]	.052	.050	[.042, .058]	.053	.050	[.042, .058]	.054	.050	[.042, .058]
20	No	3000	S	.053	.050	[.042, .058]	.058	.050	[.042, .058]	.058*	.050	[.042, .058]	.058	.050	[.042, .058]
20	Small	100	S	.004*	.092	[.081, .103]	.108*	.093	[.082, .103]	.105*	.093	[.082, .103]	.165*	.093	[.082, .103]
20	Small	250	S	.085*	.179	[.165, .193]	.193	.181	[.167, .195]	.189	.181	[.167, .195]	.215*	.182	[.168, .196]
20	Small	500	S	.296*	.366	[.348, .383]	.386	.372	[.354, .390]	.384	.372	[.354, .390]	.401*	.373	[.355, .390]
20	Small	1000	S	.708*	.726	[.710, .742]	.742	.734	[.718, .750]	.740	.734	[.718, .751]	.748	.735	[.719, .751]
20	Small	3000	S	1.000	.999	[.999, 1.000]	1.000	1.000	[.999, 1.000]	1.000	1.000	[.999, 1.000]	1.000	1.000	[.999, 1.000]
20	Large	100	S	.019*	.161	[.148, .174]	.195*	.167	[.154, .181]	.192*	.167	[.154, .181]	.255*	.168	[.155, .182]
20	Large	250	S	.274*	.411	[.393, .429]	.443	.431	[.413, .449]	.439	.431	[.413, .449]	.479*	.434	[.416, .452]
20	Large	500	S	.727*	.785	[.770, .800]	.800	.807	[.793, .822]	.796	.807	[.793, .822]	.806	.811	[.797, .825]
20	Large	1000	S	.987*	.991	[.987, .994]	.991	.993	[.991, .996]	.991	.993	[.991, .996]	.992	.994	[.991, .997]
20	Large	3000	S	1.000	1.000	[1.000, 1.000]	1.000	1.000	[1.000, 1.000]	1.000	1.000	[1.000, 1.000]	1.000	1.000	[1.000, 1.000]
50	No	100	S	.000*	.050	[.042, .058]	.075*	.050	[.042, .058]	.102*	.050	[.042, .058]	.136*	.050	[.042, .058]
50	No	250	S	.003*	.050	[.042, .058]	.062*	.050	[.042, .058]	.081*	.050	[.042, .058]	.078*	.050	[.042, .058]
50	No	500	S	.014*	.050	[.042, .058]	.056	.050	[.042, .058]	.079*	.050	[.042, .058]	.065*	.050	[.042, .058]
50	No	1000	S	.026*	.050	[.042, .058]	.054	.050	[.042, .058]	.081*	.050	[.042, .058]	.054	.050	[.042, .058]
50	No	3000	S	.045	.050	[.042, .058]	.059*	.050	[.042, .058]	.096*	.050	[.042, .058]	.058	.050	[.042, .058]
50	Small	100	S	.000*	.070	[.060, .079]	.091*	.070	[.061, .080]	.120*	.071	[.062, .081]	.162*	.070	[.061, .080]
50	Small	250	S	.009*	.108	[.096, .119]	.119	.109	[.098, .121]	.154*	.112	[.101, .124]	.148*	.109	[.097, .120]
50	Small	500	S	.077*	.192	[.177, .206]	.191	.196	[.181, .210]	.242*	.204	[.189, .218]	.211*	.195	[.180, .209]
50	Small	1000	S	.308*	.415	[.397, .433]	.406*	.425	[.407, .443]	.473*	.444	[.426, .462]	.413	.422	[.404, .440]
50	Small	3000	S	.948*	.968	[.961, .974]	.957*	.972	[.965, .978]	.973	.978	[.972, .983]	.957*	.971	[.964, .977]
50	Large	100	S	.000*	.100	[.089, .111]	.132*	.102	[.091, .114]	.165*	.103	[.092, .114]	.217*	.102	[.091, .114]
50	Large	250	S	.028*	.213	[.198, .228]	.233	.222	[.207, .237]	.279*	.224	[.209, .240]	.274*	.222	[.207, .237]
50	Large	500	S	.268*	.467	[.448, .485]	.471	.487	[.469, .505]	.522*	.493	[.475, .511]	.494	.488	[.469, .506]
50	Large	1000	S	.787*	.867	[.855, .880]	.855*	.885	[.873, .896]	.883	.889	[.878, .901]	.859*	.885	[.873, .897]
50	Large	3000	S	1.000	1.000	[1.000, 1.000]	1.000	1.000	[1.000, 1.000]	1.000	1.000	[1.000, 1.000]	1.000	1.000	[1.000, 1.000]

*A* analytical method, *S* sampling-based method, *n* sample size, *OHR* observed hit rate, *EHR* expected hit rate, *Env* 99% confidence envelope of the expected hit rate. OHR’s that lie outside of the envelope are marked with a star.
