# Supplementary material for: Power Analysis for the Wald, LR, Score, and Gradient Tests in a Marginal Maximum Likelihood Framework: Applications in IRT
Source: Psychometrika. 2022 Aug 27;88(4):1249–98. doi: 10.1007/s11336-022-09883-5 (PMC10656348; doi:10.1007/s11336-022-09883-5)
Supplement: Supplementary file 1 — (zip 115543 KB) [file 11336_2022_9883_MOESM1_ESM.zip › vignettes/adding_hypotheses.html]

Adding Hypotheses


# Adding Hypotheses

- Walkthrough
  - Function to set up the restricted model
  - Function to set up the unrestricted model
  - Function to maximize the likelihood of the restricted parameters
  - Putting it together
- Testing the new hypothesis

```
library(pwrml)
library(mirt)
library(dplyr)
```

# Walkthrough

This vignette introduces the addition of new hypothesis to allow for a power analysis for the Wald, LR, score and gradient test. It is intended for advanced R users. The necessary steps are:

- Function to set up the restricted model
- Function to set up the unrestricted model
- Function to maximize the likelihood of the restricted parameters
- Putting it together

As an example, we will set up a simple hypothesis, testing whether the difficulty of the first item is equal to 0.

## Function to set up the restricted model

In our restricted model, we define the A Matrix and the c vector. They formally represent the hypothesis. Then we setup the instructions for mirt to fit the model, which imply to keep the first item difficulty fixed at 0.

```
  res = function(altpars,nullpars = NULL) {

    n.items = length(altpars[[1]])

    re = list(
      n.items = n.items,
      itemtype = "2PL",
      Amat = c(0,1,rep(0,(n.items-1)*2)) %>% matrix(.,ncol=n.items*2,byrow=TRUE),
      cvec = 0,
      model = mirt::mirt.model(paste('F = 1-',n.items,'
                           FIXED = (1, d)
                           START = (1,d,0)'))
    )
    return(re)
  }
```

## Function to set up the unrestricted model

The unrestricted model is a basic 2PL model. Note that we generate a longpars object that represents beta in the multiplication “A \* beta = c”. It results from a concatenation of the discrimination and difficulty parameters.

```
unres = function(altpars) {

    re = list(
      parsets = altpars,
      model = 1,
      itemtype = "2PL",
      longpars = pars.long(pars = altpars,itemtype="2PL")
    )

    return(re)
  }
```

## Function to maximize the likelihood of the restricted parameters

We define a function that calculates the maximum likelihood parameters under the restricted model. In this specific case, we know that the restricted model parameters can be expected to result in the true parameters for all but the first item. The only parameter in question is the discrimination of the first item parameter. We therefore ask the question: If we set the first item difficulty to 0, what is the most likely item discrimination given the data follows the true parameters.

```
  maximizeL = function(hyp) {
    # Hypothesis-specific algorithm to find the maximum likelihood restricted parameter set

    maxlpreload= function(pars) {
      # returns the density for each response pattern under the model parameters pars

      patterns = as.matrix(expand.grid(lapply(1:length(pars$a),function(x) c(0,1))))

      pre = c()
      for (i in 1:nrow(patterns)) {
        pre[i] = g(patterns[i,],pars)
      }

      return(pre)
    }

    maxl = function(x,pars,pre) {
      # calculates the likelihood of parameters x given model "pars"
      patterns = as.matrix(expand.grid(lapply(1:length(pars$a),function(x) c(0,1))))

      x = list(
        a=c(x,pars$a[2:length(pars$a)]),
        d=c(0,pars$d[2:length(pars$d)])
        )

      res  = c()
      for (i in 1:nrow(patterns)) {
        px = pre[i]
        qx = g(patterns[i,],x)
        res[i] =  {px*log(qx)}
      }
      re = -sum(res)
    }
    resmod = hyp$resmod
    unresmod = hyp$unresmod

    pars = unresmod$parsets
    load.functions(unresmod$itemtype)

    startval = pars$a[1]

    maxlpre = maxlpreload(pars)

    optpar = optim(startval,function(x) {maxl(x,pars,maxlpre)},method = "BFGS")
    re = pars
    re$a = c(optpar$par[1],pars$a[2:length(pars$a)])
    re$d = c(0,pars$d[2:length(pars$d)])

    return(re)
  }
```

## Putting it together

We simply put the functions defined above in a list.

```
h_basic = list(
  res = res,
  unres = unres,
  maximizeL = maximizeL
)
```

# Testing the new hypothesis

The new hypothesis can be set up using the h\_basic list object as the type argument of the setup\_hypothesis function. We may then calculate the power at arbitrary sample sizes.

```
altpars <- list(
        a = rlnorm(5,sdlog = .4),
        d = rnorm(5)
        )

altpars$d[1]=.2

hyp <- setup_hypothesis(type = h_basic, altpars = altpars)

ncps <- calculate_ncps(hyp=hyp)

n = seq(100,2000)
pow = power(hyp=hyp,ncp=ncps["Gradient"],alpha=.05,ssize=n)
plot(n,pow)
```
