# Supplementary material for: Power Analysis for the Wald, LR, Score, and Gradient Tests in a Marginal Maximum Likelihood Framework: Applications in IRT
Source: Psychometrika. 2022 Aug 27;88(4):1249–98. doi: 10.1007/s11336-022-09883-5 (PMC10656348; doi:10.1007/s11336-022-09883-5)
Supplement: Supplementary file 1 — (zip 115543 KB) [file 11336_2022_9883_MOESM1_ESM.zip › vignettes/demo.html]

Demo


# Demo

- Introduction
- Workflow example
- Functions in detail
  - Setup Hypotheses (setup\_hypothesis)
  - Calculate Noncentrality Parameters (calculate\_ncps)
  - Calculate Power or Sample Size (power, ssize)
- Additional Features
  - Generate artificial datasets
  - Performing Hypothesis Tests
  - Estimating the time needed for the analytical approach
- Further Examples
  - 3PL model
  - Multidimensional model

```
library(pwrml)
library(mirt)
library(dplyr)
```

# Introduction

This vignette introduces the implementation of a power analysis for the Wald, LR, score and gradient test for linear hypothesis. It uses some IRT examples and treats basic as well as additional features of the package. It is directed towards beginner to intermediate level R users. Previous experience with the mirt package may be helpful. In the following we will cover the three-step workflow in detail:

- Setup Hypotheses (setup\_hypothesis)
- Calculate Noncentrality Parameters (calculate\_ncps)
- Calculate Power or Sample Size (power, ssize)

As additional features, we cover:

- Generate artificial datasets
- Performing hypothesis tests
- Estimating the time needed

Finally, we provide some additional models to facilitate adoption of the package for more complex hypotheses:

- 3PL model
- Multidimensional model

# Workflow example

We want to know the power and required sample size for a test of the Rasch vs 2PL model. We use the LSAT 7 Dataset from the mirt package.

As a first step, we load the dataset and fit a 2PL model.

```
dat <- expand.table(LSAT7)
mirtfit <- mirt(dat,1,verbose = FALSE)
```

The 2PL parameters are then used as parameters for the alternative hypothesis in our hypothesis definition.

```
hyp <- setup_hypothesis(type = "1PLvs2PL", altpars = mirtfit)
```

We can now calculate the noncentrality parameters.

```
ncps <- calculate_ncps(hyp=hyp)
```

From the noncentrality parameters, we can estimate the power or required sample size.

```
power(hyp=hyp,ncp=ncps,alpha=.05,ssize=500)
```

```
##      Wald        LR     Score  Gradient 
## 0.4159277 0.5218344 0.4989753 0.5841164
```

```
ssize(hyp=hyp,ncp=ncps,alpha=.05,power=.80)
```

```
##     Wald       LR    Score Gradient 
##     1134      887      932      778
```

# Functions in detail

We will present all included functions and their arguments in more detail.

## Setup Hypotheses (setup\_hypothesis)

We can either use a fitted mirt model or specify alternative parameters directly. An example of the letter would be:

```
altpars <- list(
        a = rlnorm(5,sdlog = .4),
        d = rnorm(5)
        )
altpars
```

```
## $a
## [1] 0.8599582 1.5704589 0.5974301 0.7690267 0.8516850
## 
## $d
## [1] -0.4715208  0.1892924  0.2369654  1.7075024 -0.7447248
```

```
hyp <- setup_hypothesis(type = "1PLvs2PL", altpars = altpars)
```

The following alternative hypotheses are currently implemented.

- Rasch against 2PL
- DIF in 2PL
- PCM against GPCM

The Rasch against 2PL hypothesis is presented above. The procedure for the DIF in 2PL hypothesis is analogous, yet is a bit more complicated since we need to define different groups:

```
group1 = group2 <- list(
        a = rlnorm(5,sdlog = .2),
        d = rnorm(5)
        )

group2$a[1] = (group2$a[1])^2
group2$d[1] = group2$d[1] + .5

altpars <- list(group1,group2)

altpars
```

```
## [[1]]
## [[1]]$a
## [1] 0.9184613 1.1851130 0.8691867 0.7902325 1.0185416
## 
## [[1]]$d
## [1]  0.63195855  1.11927904  0.36057828 -0.07420291  0.27239791
## 
## 
## [[2]]
## [[2]]$a
## [1] 0.8435711 1.1851130 0.8691867 0.7902325 1.0185416
## 
## [[2]]$d
## [1]  1.13195855  1.11927904  0.36057828 -0.07420291  0.27239791
```

```
hyp <- setup_hypothesis(type = "DIF2PL", altpars = altpars)
```

To implement custom hypotheses, please refer to the respective vignette. Note that for both hypothesis types, we do not have to provide the parameters under the null hypothesis here, because they are implicitly defined by the hypotheses. The setup\_hypothesis function, however, also takes a nullpars argument for cases in which the parmaters under the null hypothesis are not identified by the parameters under the alternative.

## Calculate Noncentrality Parameters (calculate\_ncps)

The calculation of noncentrality parameters is straightforward.

```
ncps <- calculate_ncps(hyp=hyp)
```

To use the sampling-based parameters, one may use sampling=TRUE. The sample size of the sampling-based approach and the approximation of the Fisher expected matrix may be tweaked or left at their default values.

```
ncps <- calculate_ncps(hyp=hyp,sampling=TRUE,sampling.npers = 10^4,approx.npers=10^4)
```

## Calculate Power or Sample Size (power, ssize)

The functions to calculate power and sample size are straightforward. One may use them to plot a power curve.

```
n = seq(100,2000)
pow = power(hyp=hyp,ncp=ncps["Gradient"],alpha=.05,ssize=n)
plot(n,pow)
```

# Additional Features

We will take a look at some additional features.

## Generate artificial datasets

The hypothesis object can also be used to generate data according to the parameters of the alternative hypothesis. Note that setup.data also allows for non-normal person distributions, e.g. uniform or skewed-normal distributions.

```
altpars <- list(
        a = rlnorm(5,sdlog = .4),
        d = rnorm(5)
        )

hyp <- setup_hypothesis(type = "1PLvs2PL", altpars = altpars)

data <- setup.data(hyp=hyp,n=500)
```

## Performing Hypothesis Tests

One can also perform hypothesis tests on observed data. This is done in two steps, model fitting (mml.fit) and calculation of the statitics (stat\_obs). To fit both an unrestricted and restricted model in one go, we specify the data and the hypothesis.

```
fitted <- mml.fit(data = data,hyp = hyp)
```

One may also use an approximation of the Fisher expected matrix, e.g. for larger item sets.

```
fitted <- mml.fit(data = data,hyp = hyp,infmat.unres = "ApproxFisher",infmat.res="ApproxFisher",approx.npers = 10^4)
```

From the results, we can calculate the observed statistics and p-values.

```
stats_obs <- stat_obs(fitted)
stats_obs
```

```
##     Wald       LR    Score Gradient 
## 9.232471 7.296524 6.812363 6.968756
```

```
pvals <- pchisq(stats_obs,df=nrow(hyp$resmod$Amat),ncp=0,lower.tail=FALSE)
pvals
```

```
##       Wald         LR      Score   Gradient 
## 0.05554433 0.12102374 0.14614252 0.13754857
```

## Estimating the time needed for the analytical approach

We can calculate the approximate time needed to calculate the analytical noncentrality parameters using a larger numbers of items.

```
altpars <- list(
        a = rlnorm(5,sdlog = .4),
        d = rnorm(5)
        )

hyp <- setup_hypothesis(type = "1PLvs2PL", altpars = altpars)

calctime(hyp,n.items=7)
```

```
## [1] 14.08
```

Measuring the actual time for comparison:

```
altpars <- list(
        a = rlnorm(7,sdlog = .4),
        d = rnorm(7)
        )

hyp <- setup_hypothesis(type = "1PLvs2PL", altpars = altpars)

calctime(hyp,n.items=7)
```

```
## [1] 14.82
```

The suggested time does deviate sometimes from the actual time. As a result from our test runs, I suggest to expect +80% as a worst case scenario.

# Further Examples

To demonstrate extensability of the method to models not treated in the paper, we demonstrate testing some basic hypotheses in the 3PL model and a multidimensional model. We present only the sampling-based method here, but it can be extended to the analytical method in the future.

## 3PL model

We test the null hypothesis that the first item parameters are (1,0,.2) for the a,d, and g parameters respectively. The parameters under the alternative hypothesis are:

```
altpars <- list(
  a = c(1.1,seq(.5,1.4,length.out=4)),
  d = c(.1,seq(1.3,-1.3,length.out=4)),
  g = c(.22,rep(.2,4))
)
altpars
```

```
## $a
## [1] 1.1 0.5 0.8 1.1 1.4
## 
## $d
## [1]  0.1000000  1.3000000  0.4333333 -0.4333333 -1.3000000
## 
## $g
## [1] 0.22 0.20 0.20 0.20 0.20
```

Calculating sampling-based noncentrality parameters

```
hyp <- setup_hypothesis(type = "3PL_basic", altpars = altpars)
set.seed(1)
ncps <- calculate_ncps(hyp=hyp,sampling=TRUE,sampling.npers = 10^5,approx.npers=10^5,SE.type="Fisher")
```

Power and sample size

```
power(hyp=hyp,ncp=ncps,alpha=.05,ssize=500)
```

```
##      Wald        LR     Score  Gradient 
## 0.1644277 0.1498761 0.1496394 0.1437990
```

```
ssize(hyp=hyp,ncp=ncps,alpha=.05,power=.50)
```

```
##     Wald       LR    Score Gradient 
##     1747     1973     1977     2087
```

## Multidimensional model

We test the null hypothesis that the difficulty of the first two item parameters is equal. The parameters under the alternative hypothesis are taken from the LSAT7 dataset:

```
dat <- expand.table(LSAT7)
dat = dat[,c(2,4,1,3,5)] # re-ordering items, items 1 and 2 of the resulting data.frame are compared
model_an <- 'F1 = 1-5
      F2 = 1-4'
altpars = mirt(dat, model = mirt.model(model_an),verbose = FALSE)
coef(altpars,simplify=T)
```

```
## $items
##           a1     a2     d g u
## Item.2 0.848  0.522 0.787 0 1
## Item.4 0.756  0.028 0.485 0 1
## Item.1 2.003 -0.866 2.644 0 1
## Item.3 2.156  1.843 2.487 0 1
## Item.5 0.758  0.000 1.864 0 1
## 
## $means
## F1 F2 
##  0  0 
## 
## $cov
##    F1 F2
## F1  1  0
## F2  0  1
```

Calculating sampling-based noncentrality parameters

```
hyp <- setup_hypothesis(type = "multi_basic", altpars = altpars)
set.seed(1)
ncps <- calculate_ncps(hyp=hyp,sampling=TRUE,sampling.npers = 10^5,approx.npers=10^5)
```

```
## EM cycles terminated after 5000 iterations.
```

Power and sample size

```
power(hyp=hyp,ncp=ncps,alpha=.05,ssize=1000)
```

```
##      Wald        LR     Score  Gradient 
## 0.6849039 0.7246045 0.7385198 0.7356491
```

```
ssize(hyp=hyp,ncp=ncps,alpha=.05,power=.80)
```

```
##     Wald       LR    Score Gradient 
##     1317     1201     1163     1171
```
